# Thermal and mechanical properties of polylactic acid reinforced with surface-treated hemp composites for automotive applications

**DOI:** 10.1177/00368504251352072

**Published:** 2025-10-16

**Authors:** Sifiso J Skosana, Moshibudi C Khoathane, Thomas Malwela, Wilson Webo

**Affiliations:** 1 Department of Chemical, Metallurgical and Materials Engineering, Faculty of Engineering and the Built Environment, Tshwane University of Technology, Pretoria, South Africa; 2 Department of Physics, 37714 University of Limpopo, Sovenga, South Africa

**Keywords:** Composites, polylactic acid, surface-treated hemp fibers, additive manufacturing, response surface methodology, and automotive

## Abstract

Thermal and mechanical properties are important in the automotive industry for manufacturing reliable and durable vehicle components. Hence, this work examines the thermal and mechanical properties of polylactic acid (PLA) reinforced with surface-treated hemp composites for automotive interior components. The composites were prepared using the additive manufacturing technique, while response surface methodology (RSM) was utilized to identify the optimal processing parameters, thus reducing wastage of material from many experimental trials. The optimum parameters were identified as: 1% volume fraction, 40 mm/s print velocity, 1 mm nozzle diameter, and 45° printing direction. These conditions together with surface treatment significantly improved the tensile elastic modulus to 2607.27 MPa and impact strength to 16.66 MPa of the composite when compared to 2353.27 and 21.01 MPa of pure PLA, respectively. Surface morphology from atomic force microscopy and scanning electron microscopy showed an increased surface roughness and complex surface topography on the composite, while differential scanning calorimetry and thermogravimetry analysis revealed an enhanced crystallization behavior and thermal stability. The composite had a sharper crystallization peak at a lower temperature and decomposed more gradually than pure PLA. This study demonstrates the effectiveness of using RSM to optimize three-dimensional printed natural fiber-reinforced polymer composite materials for interior automotive components, achieving a good desirability function analysis score of 0.782.

## Introduction

The automotive industry is transforming towards sustainable and eco-conscious practices driven by increasing environmental concerns and stringent emission regulations.^1–4^ In this regard, natural fiber-reinforced polymer composites (NFRPCs) have emerged as a promising solution for eco-friendly lightweight application.^5–8^ These composites combine natural fibers derived from renewable sources, such as hemp, flax, and jute, with polymer matrices, offering a unique combination of lightweight, renewability, and biodegradability.^9^ Among the various natural fibers, hemp fibers exhibit an impressive combination of high tensile strength and low density, rendering them a compelling reinforcement option for NFRPCs. Their eco-friendly nature further underscores their suitability for sustainable applications.^10,11^ Moreover, the ability of biodegradable plastics and natural fibers to decompose through the action of living organisms offers a practical solution to reducing the 60% of all plastics that end up in landfills due to their non-recyclable nature.^12^

Previous studies have demonstrated the potential of hemp fiber-reinforced composites in applications demanding high-impact strength and hardness.^13^ Dolçà et al.^14^ reported a significant increase in Young's modulus and impact strength of about 5275 MPa and 3.6 kJ/m^2^ after the addition of 40% weight loading of hemp, respectively, in comparison to those of pure polylactic acid (PLA) 826 MPa and 2.0 kJ/m^2^. In another study Studies by Murugu et al.^13^ and Sullins et al.^10^ have demonstrated the potential of hemp fiber composites in applications requiring high-impact strength and hardness, highlighting the effectiveness of material treatments in improving bonding between hemp fibers and polymer matrices. Also, research by Lee et al.^15^ has underscored hemp fiber's competitive advantage over traditional materials like fiberglass, particularly in terms of cost, density, and mechanical qualities.

This positions hemp fiber as a sustainable substitute in the automotive sector, aligning with the industry's increasing focus on eco-conscious practices and regulatory demands to reduce emissions by light-weighting car components and promote sustainability. The integration of natural fibers in polymer matrices not only enhances mechanical properties but also reduces reliance on non-renewable resources. NFRPCs exhibit high water absorption and are limited in their long-term use for applications in humid environments. However, when good bonding between the fibers and the polymer matrix is achieved, NFRPCs are less prone to oxygen attack and moisture-induced degradation. They are more suitable for applications that do not involve high temperatures or humidity, such as automotive interiors.^16^ Surface treatments, such as chemical modification of hemp fibers and the addition of compatibilizers, have been shown to improve the interfacial adhesion between hemp fibers and polymer matrices, resulting in composites with significantly enhanced mechanical properties.^17,18^ Alao et al.^19^ reported improvements in flexural properties of about 13%, by using alkali-treated hemp fiber without any compatibilizers. These improvements were attributed to increased surface roughness resulting from the removal of hemicellulose and lignin. Hemp fibers are composed of lignin, hemicellulose and cellulose constituents, and hemicellulose is the weakest. Surface treatment reduces the content of hemicellulose and lignin, thereby increasing the degree of crystalline packing order and surface roughness. This enhances the mechanical interlocking, wetting of the fiber by the polymer and increase fiber-matrix interfacial adhesion, allowing for more efficient stress transfer between the matrix and fibers.^13,20,21^

Most researchers have used a 5 wt% NaOH concentration as a moderate treatment for hemp fiber because lower concentrations result in incomplete removal of hemicellulose, pectin, and lignin, while higher concentrations are too harsh and can damage the fibers, leading to chain scission.^10,19,22–24^ Mwaikambo and Ansell^25^ reported that alkali treatment reduces the cellulose content, which in turn increases the tensile strength and Young's modulus due to enhanced crystalline packing order, indicating that the packing order influences the mechanical properties. Sawpan et al.^24^ reported a substantial increase in impact strength for composites incorporating treated hemp fibers. This enhancement of the composite materials total impact resistance can be ascribed to the natural fibers capacity to absorb energy, enhanced PLA matrix crystallinity and withstand fractures.

Among the processing techniques, additive manufacturing, commonly referred to as three-dimensional (3D) printing, represents an emerging frontier for fabricating NFRPC components. This process enables the creation of intricate and customized designs while minimizing material waste. However, challenges persist in achieving high fiber volume fractions (Vfs) and maintaining desired mechanical performance. Higher Vfs has been found to increase the mechanical properties of the NFRPCs, however, they also lead flow issues, poor printability, and weak interfacial bonding due to the formation of agglomerations.^26–29^ Some researchers attempt to change the fiber type to maximize the Vf; however, this approach does not always enhance the material properties and may introduce other limitations, necessitating further research and optimization.^30^ Moreover, Ning et al.^31^ found that the mechanical properties of polymer composites were significantly influenced by factors such as raster angle, infill speed, nozzle temperature, and layer thickness. Advancements in research domains have identified response surface methodology (RSM) as one of the most significant and convenient techniques for investigating the interactions of multiple independent variables in the processing and manufacturing of polymer composites, thereby reducing material wastage and time compared to conducting numerous trials.^32,33^ Dash et al.^34^ utilized RSM to determine the optimal operating conditions while investigating the mechanical and tribological performance of hybrid composites. Their findings revealed a 3% reduction in surface wear rate under optimized parameters. Haniel et al.^35^ reported an average of 0.241 J/mm^2^ impact strength and 94.8650 MPa flexural strenghth, when using optimized values derived from RSM.

In this context, the present study aims to optimize the thermal and mechanical performance of PLA reinforced with surface-treated hemp composite for automotive applications using RSM-optimized conditions such as Vf, printing velocity, nozzle diameter and printing direction (raster angle). PLA, a biodegradable and renewable polymer derived from lactic acid, offers a sustainable alternative to conventional petroleum-based plastics.^36^ PLA is also cost-effective, less sensitive to humidity and has a wide temperature range, making easier to print in comparison to other biopolymers such as polyhydroxyalkanoates, cellulose and polybutylene adipate terephthalate.^37^ Composites made out of PLA offer potential as replacements for existing interior automotive components currently manufactured from synthetic fiber and petroleum-based polymers such as dashboards, hat racks, spare tire linings, seat parts, and boot linings.^38^ By incorporating surface-treated hemp fibers into the PLA matrix, the study seeks to enhance the mechanical and thermal properties of the resulting composite, thereby addressing the growing demand for high-performance, eco-friendly materials in the automotive industry.^39,40^

To achieve this objective, the research employs a systematic approach utilizing additive manufacturing techniques and response surface design methodology. The integration of additive manufacturing enables the fabrication of complex geometries and the exploration of various process parameters, while the response surface design methodology facilitates the optimization of the composite's mechanical and thermal properties by evaluating the influence of multiple factors and their interactions.

## Experimental section

### Materials

The matrix employed in the extrusion process was a commercial grade PLA sourced from Total Energies Corbion under the trade name LUMINY^®^ LX175. The PLA exhibited an L-isomer content of approximately 96% and a melt flow index (MFI) of 6 g/10 min. The reinforcing Hemp fibers, with average lengths ranging from 0.5 to 1 cm, were generously donated by the former CSIR Gqeberha Textile laboratory. Analytical research (AR) grade acetone and sodium hydroxide (NaOH) were acquired from Sigma Aldrich and utilized without further purification. Throughout the experiments, deionized water served as the solvent.

### Fabrication of 3D printed PLA reinforced with surface-treated HEMP composite

#### Fiber treatment

The fibers underwent a meticulous treatment process to ensure optimal adhesion and compatibility with the PLA matrix. Initially, the fibers were washed with water to eliminate any soil contaminants and subsequently dried in an air oven at 60 °C for 24 h. Following onto this, they were immersed in 1000 ml glass beaker containers containing a 5% w/v NaOH solution. The fibers underwent a 24-h soaking period at room temperature while being continuously stirred at 150 r/min with a mechanical stirrer. Subsequently, the treated fibers were rinsed with acetone and deionized water multiple times to remove any absorbed alkali before undergoing another 24-h drying period in an air oven at 60 °C.

#### Filament extrusion

The PLA matrix and treated hemp fibers were subjected to a drying process in an air oven at 60 °C for 24 h to eliminate any absorbed moisture prior to processing. Filament production occurred using a Process 11 twin-screw extruder from Thermos Scientific, Waltham, MA, USA, with an L/D (length to diameter) ratio of 40. The fiber loading ranged from 1% to 20% weight percent (w/w). The temperature profile for all samples was carefully controlled, ranging from 170 to 190 °C, with a feeding rate of 10 kg/h and screw speed set at 220 r/min. After extrusion, the material was pelletized and underwent another 24-h drying process. Subsequently, the pellets were subjected to a second extrusion under identical conditions to achieve homogeneous distribution of the fibers in the polymer matric. Filaments with a formulated diameter of 1.75 mm were produced by extruding the polymer composites using a FelFil-Evo small 3D filament maker with an L/D ratio of 12.7. The operating temperature and screw speed were set to 190 °C and 6 r/min, respectively. A 1.75 mm nozzle was used to achieve the desired filament thickness, and the filament was air-cooled as it was drawn onto the spool.

#### Fused deposition modeling

Test specimens were fabricated with fused deposition modeling (FDM) technique using a Creality CR-10s from Shenzhen, China. Ultimaker Cura software was employed to slice the CAD drawings of the specimens for printing. A flat-head nozzle was utilized to drive and compress the filament during printing, with nozzle sizes ranging from approximately 0.4 to 2 mm. The temperature of the nozzle and heating bed plate was maintained at a constant 210 °C and 70 °C, respectively. The raster angles varied during printing, with orientations set at 0°, 45°, and 90° for short fibers. The fill pattern and printing speed were set at 100% and 60 mm/s, respectively.

### Response surface methodology

RSM offers an effective approach to reducing experimental costs by employing streamlined versions of complex models known as response surfaces or metamodels. These surfaces approximate the input-output relationship of underlying simulation models, aiding in the optimization of processes and system performance. RSM is particularly valuable when dealing with scenarios where a small number of unrelated input factors significantly influence the response of interest.

Given the inherent uncertainty in actual response functions, accurately approximating and regulating specific parameters describing response quality is critical in RSM. The methodology provides immediate output parameters through approximate evaluation, minimizing computational efforts. Moreover, with only a few design points, RSM can achieve high precision for the response function, with various metrics employed to regulate the quality of the response surface.

In this study, the Design Expert 13 program facilitated RSM implementation, with the optimal (custom) design chosen from alternative designs including central composite and Box-Behnken designs. The selection of run parameters was algorithmically optimized to enhance the thermal and mechanical performance of PLA reinforced with surface-treated HEMP composite for automotive applications while minimizing the number of experimental runs (Table 1).

**Table 1.** Design of experiments and corresponding response variables for polylactic acid (PLA)/surface-treated hemp fiber composites.

	Factor 1	Factor 2	Factor 3	Factor 4	Response 1	Response 2	Response 3
Run	A: Volume fraction	B: Print velocity	C: Nozzle diameter	D: Printing direction	Elastic modulus	Impact strength	Thermal degradation temperature
	%	mm/s	mm	Â°	MPa	kJ/m^2^	°C
1	4.8	55.1	1	45	2201.2	25.14	322.67
2	14.205	54.7968	1	0	2163.3	15.47	328.67
3	15.44	44	0.4	45	2112.3	10.65	293.50
4	20	52	2	0	1936.4	6.11	315.33
5	20	46.5	2	45	2524.6	7.85	313.33
6	19.05	60	0.4	0	2189.9	6.12	292.17
7	1	40	1	0	1954.6	14.55	335.83
8	4.8	55.1	1	45	2127.4	24.95	320.85
9	8.79	40	2	0	2189.4	19.31	308.50
10	1	48	2	90	1963.4	13.23	332.36
11	18.1	58	2	45	2643.8	7.33	293.83
12	14.205	54.7968	1	0	1896.8	16.9	328.67
13	20	40	0.4	0	2079.0	5.37	313.33
14	1	60	2	0	2154.6	16.35	328.35
15	1	60	1	90	1763.3	12.98	330.83
16	20	60	1	90	2136.4	6.11	312.33
17	12.115	60	2	90	1961.8	10.11	320.17
18	20	40	1	45	3275.3	8.25	309.26
19	15.44	44	0.4	45	2259.5	9.65	293.50
20	15.25	53.8	0.4	90	2387.0	7.33	292.50
21	3.27302	50.6025	0.4	0	2140.1	16.9	328.17
22	13.777	49	0.4	0	1562.1	5.12	301.33
23	1	46.4	0.4	45	1660.6	16.23	352.50
24	20	52.4	1	45	2374.6	7.69	316.63
25	4.99	40	0.4	0	1866.0	14.4	349.67
26	15.25	53.8	0.4	90	2423.2	8.16	296.01
27	11.45	42.7	1	90	2628.8	7.12	314.83
28	11.45	42.7	1	90	2628.8	7.12	314.83
29	2.9	42	2	45	2645.3	20.63	339.67
30	1	40	0.4	90	2699.7	12.32	334.17
31	1	60	0.4	45	1485.5	14.55	328.17
32	20	40	2	90	2136.4	8.25	308.67

Factors include volume fraction (A), print velocity (B), nozzle diameter (C), and printing direction (D). Responses measured are elastic modulus, impact strength, and thermal degradation temperature.

However, flexibility in design may incur associated costs, potentially resulting in different designs with similar characteristics.

Following the creation of the custom design, all pertinent factors for RSM analysis, including Vf (%), print velocity (mm/s), nozzle diameter (mm), and printing direction, were input into the models (Tables S1 and S3). The design model utilized in RSM was quadratic, and the experiment design type was I-optimal with Coordinate Exchange. With 32 runs conducted, responses included elastic modulus (MPa), impact strength (kJ/m^2^), and thermal degradation temperature (°C) (Tables S2 and S3), facilitating the exploration and optimization of PLA composite performance for automotive applications.^41,42^

### Characterization of 3D printed PLA reinforced with surface-treated HEMP composite

#### Morphological characterization

Surface morphologies of the NFRPCs were studied through scanning electron microscopy (SEM) using an AURIGA^®^ CrossBeam^®^ Workstation from Carl Zeiss, Oberkochen, Germany. Dog-bone-shaped samples were conditioned in an air oven at 60 °C for 24 h and subsequently subjected to freeze-dry in liquid nitrogen fracture to view the cross-section. The cryogenically fractured surfaces were sputter-coated with a palladium alloy to prevent charging before imaging. Further analysis on the surface roughness was done using WSxM 5.0 software for surface analysis and AFM.^43^

#### Thermal test: Thermogravimetric analysis

Thermal properties of the composites were analysed using a PerkinElmer thermogravimetry analysis (TGA) 4000 instrument, from Shelton, CT, USA. The NFRPC samples were tested at a heating rate of 10 °C/min from room temperature to 900 °C under a nitrogen atmosphere with a flow rate of 60 mL/min. The sample weight was maintained at 18 ± 0.5 mg, and the results were analysed using Pyris data analysis software.

#### Mechanical test

##### Tensile test

Tensile tests were conducted on 3D-printed specimens to determine elastic modulus, yield strength, and elongation-at-break using a Lloyd's EZ50^®^ testing machine from Largo, Fl, USA. Equipped with a 5 kN load cell, following ASTM D638-14 standards. Each sample had an area of 3.5 mm × 7 mm, with a test gauge length of 50 mm. Testing was performed under tension mode at a constant strain rate of 50 mm/min at room temperature following conditioning in an air oven at 80 °C for 24 h.

##### Charpy impact test

Charpy impact tests were conducted on 3D printed specimens with dimensions of approximately 80 mm × 10 mm × 4 mm (length × width × breadth) in accordance with ISO 179 standards. The tests were performed on unnotched specimens using the CEAST Pendulum Resil Impactor II from Turin, Italy. The tests were carried out at room temperature, velocity was set at 3.7 m/s, with a hammer energy of 7.5 J, and the span between supports fixed at 40 mm.

## Results and discussion

### Response surface methodology

#### Elastic modulus analysis

The elastic modulus of PLA/HEMP composites was analyzed using RSM to evaluate the influence of four key factors: Vf (A), print velocity (B), nozzle diameter (C), and printing direction (D). A reduced quadratic model (equation (1)) was developed, and analysis of variance (ANOVA) results (Table 2) confirmed the statistical significance of the model (*F* = 6.99, *p* = 0.0002), with an *R*² of 0.8676. However, the discrepancy between predicted (*R*² = 0.4292) and adjusted *R*² (0.7434), alongside a significant lack of fit (*p* = 0.0495), indicated potential limitations in predicting extreme conditions.

**Table 2.** ANOVA results for elastic modulus of PLA/surface-treated hemp fiber composites.

Source	Sum of squares	df	Mean square	*F*-value	*p*-value	
Model	3.713 × 10^06^	15	2.475 × 10^05^	6.99	0.0002	Significant
A-volume fraction	1.619 × 10^05^	1	1.619 × 10^05^	4.57	0.0483	
B-print velocity	5.780 × 10^05^	1	5.780 × 10^05^	16.32	0.0009	
C-nozzle diameter	2.270 × 10^05^	2	1.135 × 10^05^	3.20	0.0676	
D-printing direction	5.286 × 10^05^	2	2.643 × 10^05^	7.46	0.0051	
BC	2.066 × 10^05^	2	1.033 × 10^05^	2.92	0.0832	
BD	4.134 × 10^05^	2	2.067 × 10^05^	5.84	0.0125	
CD	1.298 × 10^06^	4	3.246 × 10^05^	9.16	0.0005	
B²	1.222 × 10^05^	1	1.222 × 10^05^	3.45	0.0818	
Residual	5.667 × 10^05^	16	35,417.95			
Lack of fit	5.170 × 10^05^	11	46,996.70	4.73	0.0495	Significant
Pure error	49,723.49	5	9944.70			
Cor total	4.279 × 10^06^	31				
Std. Dev.	188.20		*R*²		0.8676	
Mean	2192.85		Adjusted *R*²		0.7434	
C.V. %	8.58		Predicted *R*²		0.4292	
			Adeq precision		12.5663	

PLA: polylactic acid; ANOVA: analysis of variance.

Hence, confirmatory experiments were performed to validate the model's predictions, particularly at the extremes of the design space (See the “Optimization, desirability, validation analysis” section).

The reduced quadratic model for impact strength was expressed as:

(1) $$\begin{array}{rl} & \mathrm{Elastic}\;\mathrm{Modulus}\;=\;+2106.49\;+\;97.30\;A\;-\;182.99\;B\;-\;89.18\;C[1]\;+\;85.22\;C[2]\\ & \;\;\;\;-\;186.12D[1]+\;122.21\;D[2]\;+\;21.79\mathrm{BC}[1]\;-\;146.83\mathrm{BC}[2]\\ & \;\;\;\;+223.45\;\mathrm{BD}[1]\;-\;162.70\;\mathrm{BD}[2]+29.21\;C[1]D[1]\\ & \;\;\;\;-\;77.66\;C[2]D[1]\;-\;360.58\;C[1]D[2]+126.26\;C[2]D[2]\\ & \;\;\;\;+169.85\;B^{2} \end{array}$$

Analysis of individual factors and their interactions revealed that Vf (A), print velocity (B), printing direction (D), the interaction between print velocity and printing direction (BD), and the interaction between nozzle diameter and printing direction (CD) are statistically significant (*p* < 0.05). Print velocity demonstrated the most substantial impact on elastic modulus, with an *F*-value of 16.32 (*p* = 0.0009), followed closely by printing direction (*F*-value = 7.46, *p* = 0.0051). This finding underscores the critical role of processing parameters and build orientation in determining the elastic properties of PLA/HEMP composites.

**Figure 1.**
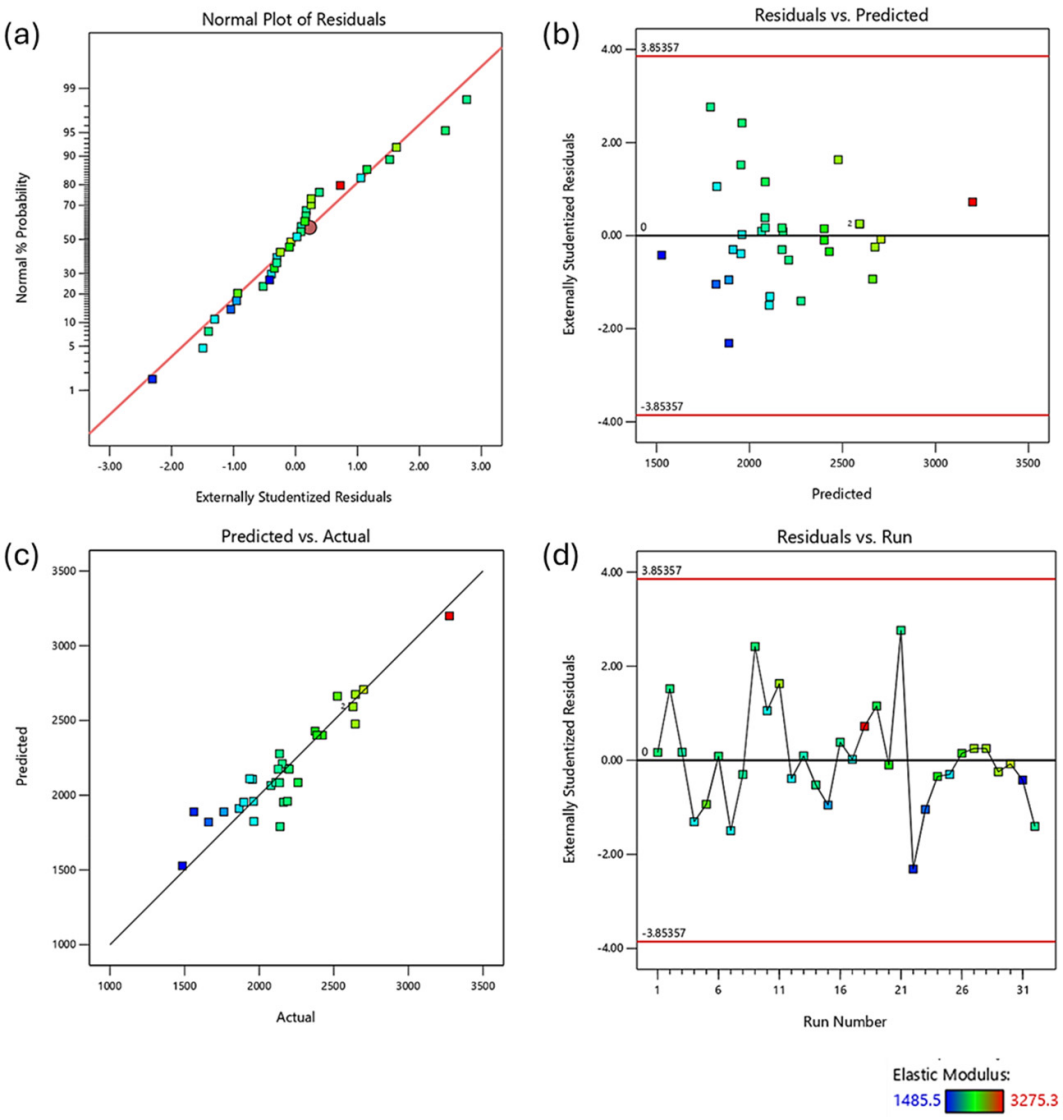
Diagnostic plots for regression analysis of elastic modulus data. (a) Normal probability plot of residuals; (b) residuals vs. predicted values; (c) predicted vs. actual values; (d) residuals vs. run number. Colors indicate elastic modulus values ranging from 1485.5 to 3275.3.

**Figure 2.**
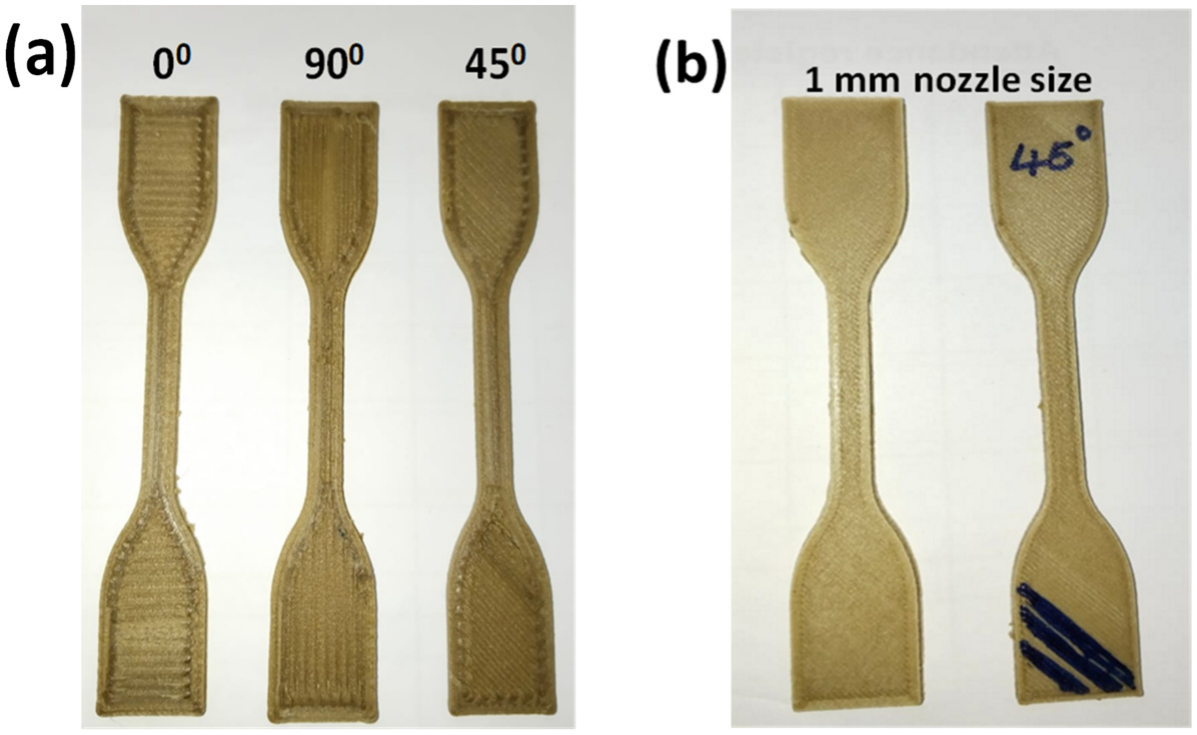
Test specimens that 3D printed at (a) different raster angles using a 2 mm nozzle size (b) printed using the 1 mm nozzle size and optimal processing conditions.

Diagnostic plots (Figure 1) were used to validate the model assumptions. The normal probability plot of residuals (Figure 1(a)) showed good adherence to normality. The residuals vs. predicted plot (Figure 2(b)) displayed a random scatter within the boundaries, indicating no obvious patterns and unequal variances. The predicted vs. actual plot (Figure 1(c)) demonstrated good correlation between predicted and measured values. The residuals vs. run plot (Figure 1(d)) showed no clear trends, suggesting the absence of time-related variables influencing the response.

**Figure 3.**
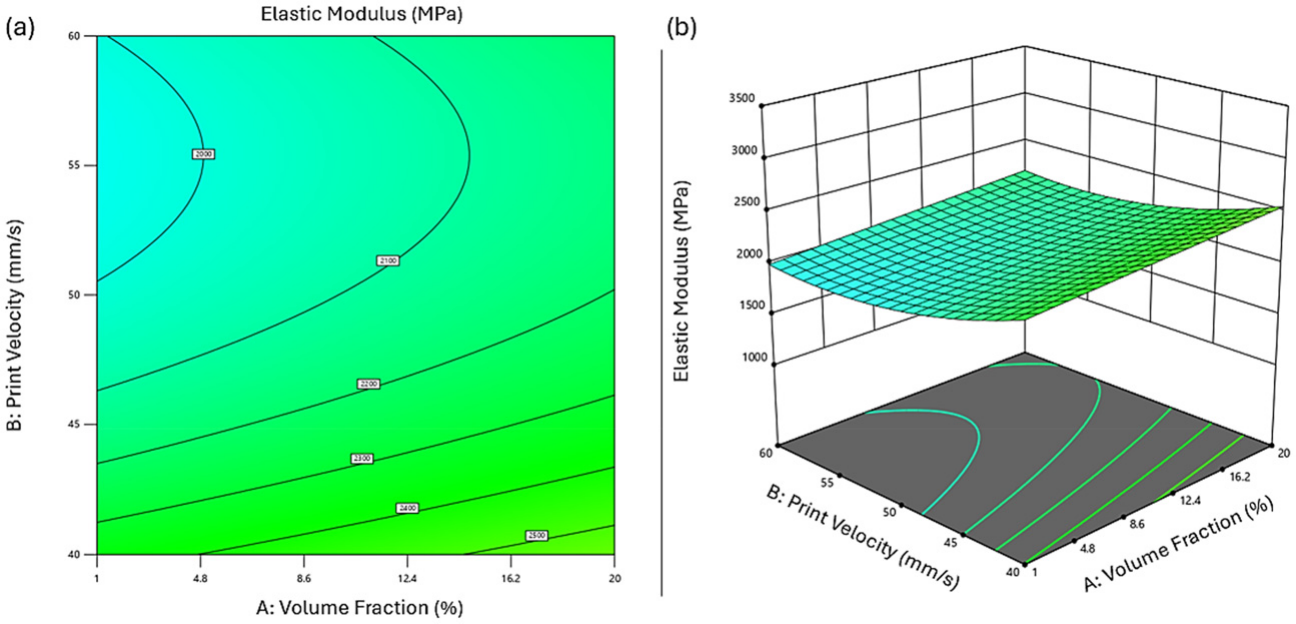
Effect of volume fraction and print velocity on elastic modulus. (a) Contour plot showing the relationship between volume fraction (%), print velocity (mm/s), and elastic modulus (MPa). (b) Three-dimensional surface plot illustrating the same relationships, with elastic modulus on the *z*-axis, print velocity on the *y*-axis, and volume fraction on the *x*-axis.

The 3D surface and contour plots (Figure 3) illustrate the combined effects of Vf and print velocity on elastic modulus. Further details of the effects of Vf (A) and print velocity (B) on the elastic modulus of PLA reinforced with surface-treated hemp composites under various processing conditions are presented in Figure S1.

Vf exhibited a direct positive correlation with modulus, as expected from the reinforcing effect of hemp fibers. Higher fiber loading enhances stiffness due to improved stress transfer at the fiber-matrix interface and the intrinsic rigidity of cellulose-rich fibers. However, the influence of print velocity was more nuanced; moderate speeds promoted better filament flow and interlayer fusion, optimizing fiber alignment and matrix continuity. Excessive print velocity (>55 mm/s) led to insufficient bonding and fiber misalignment, reducing modulus.

Nozzle diameter interacted significantly with raster angle. Smaller nozzles (e.g. 0.4 mm) led to higher polymer shear and better fiber dispersion, but also increased the risk of fiber breakage or inconsistent extrusion at higher speeds. On the other hand, larger nozzles facilitated smoother extrusion but reduced shear-induced alignment. The observed trade-offs indicate that the nozzle size must be optimized in tandem with raster orientation.

Notably, raster angle (D) had a substantial influence, with 45° orientations contributing to enhanced stiffness. This can be attributed to the quasi-isotropic fiber alignment resulting from diagonal layering, which enables load transfer in multiple directions and improves bonding between layers. In contrast, 0° and 90° orientations favored stiffness only along specific axes and led to anisotropic behavior, consistent with prior work by Iyer et al.^44^

The interaction between print velocity and raster angle (BD) further demonstrated the anisotropic nature of FDM-printed composites. At 45°, moderate speeds ensured uniform deposition and fiber-matrix interaction, while extreme speeds disrupted structural consistency. These findings underscore the critical role of processing synergy in achieving tailored mechanical performance.

#### Impact strength analysis

The impact strength of PLA/HEMP composites was model using RSM with the same input parameters. A reduced quadratic model (equation (2)) exhibited strong predictive capability (*R*² = 0.9396) and ANOVA results (Table 3) confirmed the statistically significant of the model (*F* = 16.59, *p* < 0.0001). The model showed good agreement between predicted and adjusted *R*² values, but the significant lack of fit (*p* = 0.0049) again suggests the need for further refinement at outlier combinations.

**Table 3.** ANOVA results for impact strength of PLA/surface-treated hemp fiber composites.

Source	Sum of aquares	df	Mean square	*F*-value	*p*-value	
Model	904.35	15	60.29	16.59	<0.0001	Significant
A-volume fraction	472.96	1	472.96	130.18	<0.0001	
B-print velocity	0.0282	1	0.0282	0.0078	0.9309	
C-nozzle diameter	85.54	2	42.77	11.77	0.0007	
D-printing direction	167.09	2	83.54	23.00	<0.0001	
AD	21.04	2	10.52	2.90	0.0845	
BC	79.71	2	39.85	10.97	0.0010	
CD	66.90	4	16.73	4.60	0.0115	
A²	46.88	1	46.88	12.90	0.0024	
Residual	58.13	16	3.63			
Lack of fit	56.24	11	5.11	13.56	0.0049	Significant
Pure error	1.88	5	0.3770			
Cor total	962.48	31				
Std. Dev.	1.91		*R*²		0.9396	
Mean	11.95		Adjusted *R*²		0.8830	
C.V. %	15.96		Predicted *R*²		0.8280	
			Adeq precision		14.9269	

PLA: polylactic acid; ANOVA: analysis of variance.

The reduced quadratic model for impact strength was expressed as:

(2) $$\begin{array}{rl} & \mathrm{Impact}\;\mathrm{Strength}=+14.18\;-\;5.12\;A+0.1136\;B\;-\;2.08\;C[1]+1.23\;C[2]\\ & \;\;\;\;\;\;\;+0.7952\;D[1]\;+2.26\;D[2]\;-0.2766\;\mathrm{AD}[1]\;-\;1.30\;\mathrm{AD}[2]\;\\ & \;\;\;\;\;\;\;\;\;\;\;\;\;\;\;\;\;\;\;\;\;\;\;\;\;\;\;\;\;\;\;\;\;\;\;\;\;\;\;\;\;\;\;\;\;\;\;-\;0.8681\;\mathrm{BC}[1]+3.10\;\mathrm{BC}[2]\;-\;0.6361\;C[1]D[1]\\ & \;\;\;\;\;\;\;\;\;\;\;\;\;\;\;\;\;\;\;\;\;\;\;\;\;\;\;\;\;\;\;\;\;\;\;\;\;\;\;\;\;\;\;+0.4996\;C[2]D[1]\;-\;1.16\;C[1]D[2]+2.13\;C[2]D[2]\;-\;3.27\;A^{2} \end{array}$$

Analysis of individual factors and their interactions revealed that Vf (A), nozzle diameter (C), printing direction (D), the interaction between print velocity and nozzle diameter (BC), the interaction between nozzle diameter and printing direction (CD), and the quadratic term of Vf (*A*²) are statistically significant (*p* < 0.05). The Vf demonstrated the most substantial impact on impact strength, with an *F*-value of 130.18 (*p* < 0.0001), highlighting its critical role in determining the mechanical properties of PLA/HEMP composites.

**Figure 4.**
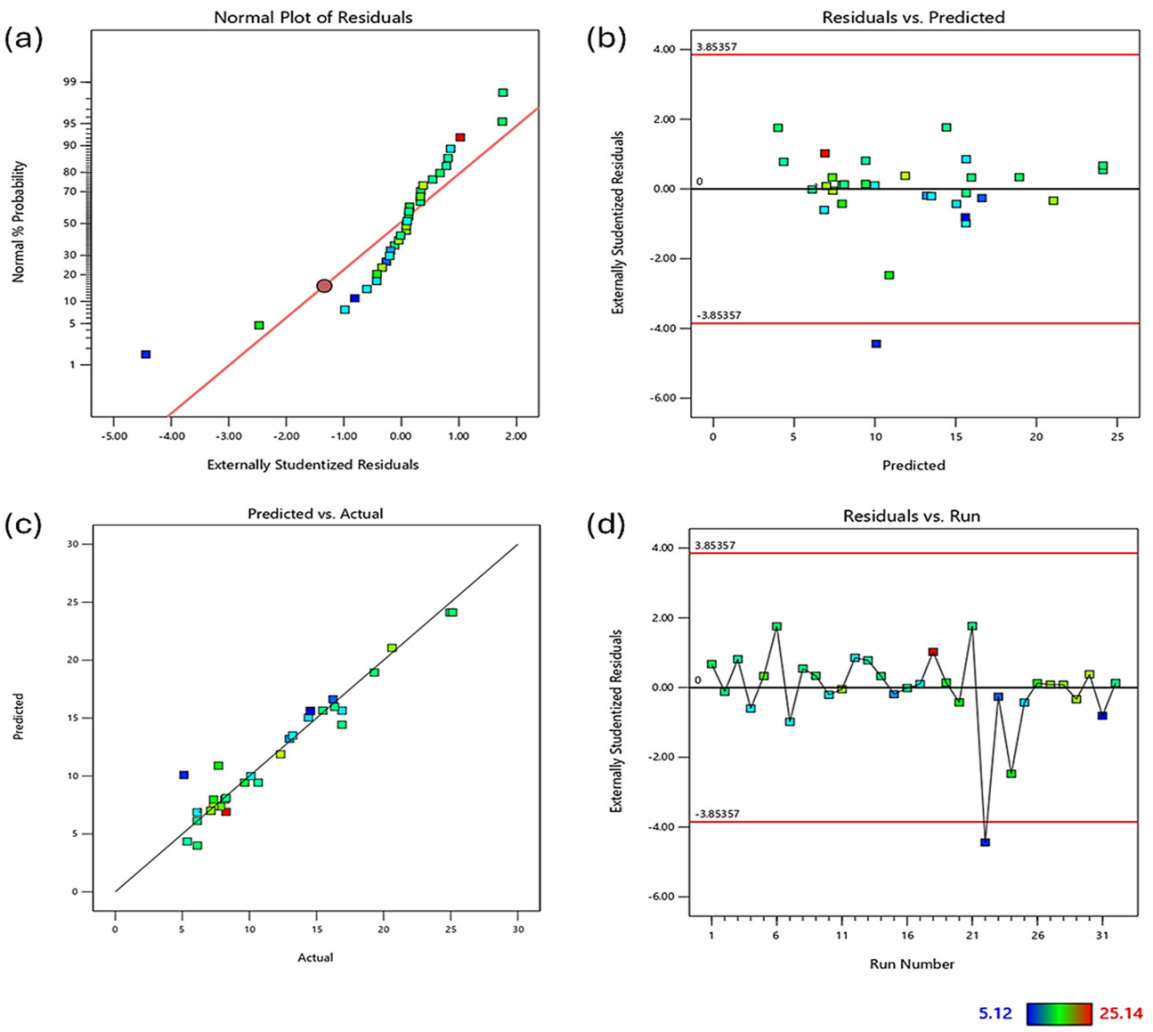
Diagnostic plots for regression analysis of impact strength of polylactic acid (PLA)-HEMP composites. (a) Normal probability plot of residuals; (b) residuals vs. predicted values; (c) predicted vs. actual values; (d) residuals vs. run number. Color scale indicates property values ranging from 5.12 to 25.14.

Diagnostic plots (Figure 4) were used to validate the model assumptions. The normal probability plot of residuals (Figure 4(a)) showed general adherence to normality, with some deviation at the extremes. The residuals vs. predicted plot (Figure 4(b)) displayed a random scatter within the boundaries, indicating no obvious patterns and unequal variances, although one potential outlier was observed. The predicted vs. actual plot (Figure 4(c)) demonstrated good correlation between predicted and measured values. The residuals vs. run plot (Figure 4(d)) showed no clear trends, suggesting the absence of time-related variables influencing the response.

**Figure 5.**
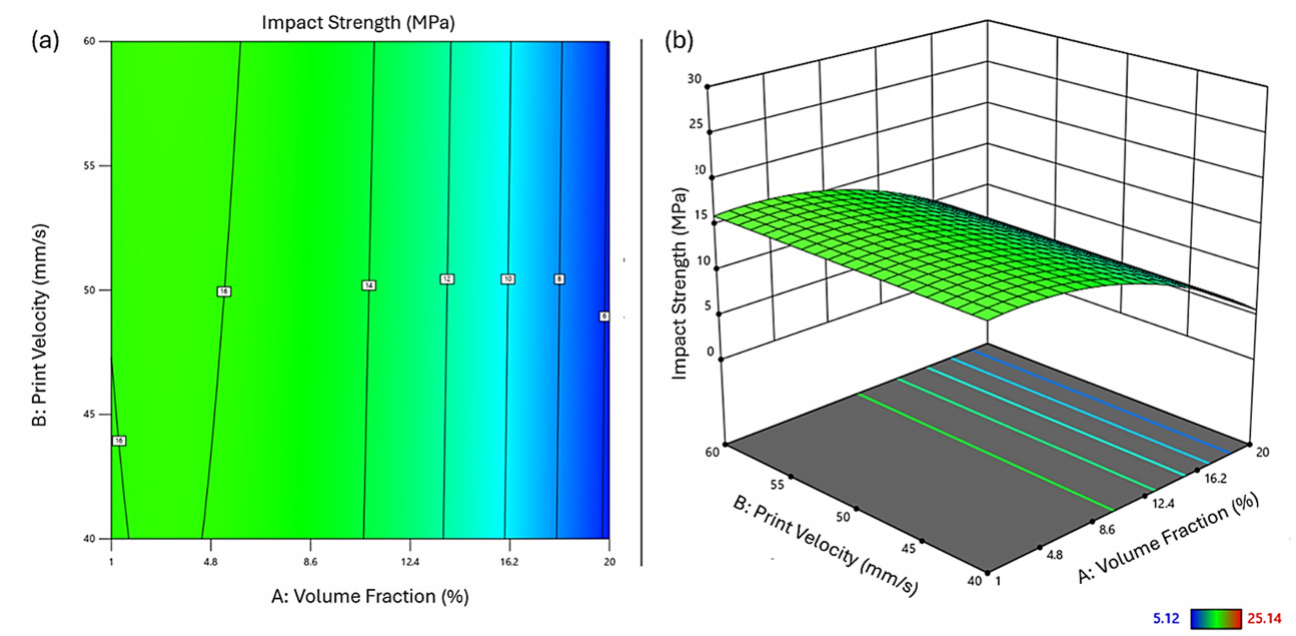
Effect of volume fraction and print velocity on impact strength of polylactic acid (PLA)-HEMP composites. (a) Contour plot illustrating the relationship between hemp volume fraction (%), print velocity (mm/s), and impact strength (kJ/m^2^). (b) Three-dimensional surface plot depicting the same relationships, with impact strength on the *z*-axis, print velocity on the *y*-axis, and hemp volume fraction on the *x*-axis. Impact strength values range from 5.12 to 25.14 kJ/m^2^ as indicated by the color scale.

The 3D surface and contour plots (Figure 5) illustrate the combined effects of Vf and print velocity on impact strength. Figure S2 illustrates how Vf (A) and print velocity (B) affect the impact strength of PLA reinforced with surface-treated hemp composites under different processing conditions. Unlike elastic modulus, impact strength decreased with increasing Vf. This inverse relationship is attributed to several factors: (1) Higher fiber content increases interfacial area and potential sites for microcracks, (2) fiber agglomeration introduces defects and stress concentrators, and (3) brittle behavior is promoted at high stiffness levels due to reduced matrix ductility. This aligns with Nigrawal et al.,^45^ who observed reduced impact resistance in over-reinforced natural fiber composites.

Print velocity showed limited direct influence but had significant interaction effects, particularly with nozzle diameter (BC). Higher velocities paired with narrow nozzles led to incomplete fusion and porosity, while moderate velocities with wider nozzles favored better fiber encapsulation and fewer voids. The combined effect improved impact resistance by facilitating energy dissipation during crack propagation.

Raster angle also played a key role. At 45°, the impact strength improved due to multidirectional stress absorption and better interlayer bonding. In contrast, 90° layers often delaminated under impact loads due to poor bonding between vertical layers. This directional dependency highlights the need to tailor build orientation based on expected loading conditions.

Furthermore, the model confirmed the nonlinear effect of fiber loading (*A*² term). Optimal impact performance was observed around 1–5% fiber content, beyond which embrittlement dominated. These insights are critical for automotive applications where components must simultaneously resist fracture and maintain lightweight properties.

#### Thermal degradation temperature

Thermal degradation behavior was also modeled using a reduced quadratic equation (equation (3)). The ANOVA results (Table 4) confirm the model was statistically significant (*F* = 8.15, *p* < 0.0001, *R*² = 0.8548), although the predicted R² (0.4778) again fell short of adjusted *R*² (0.7500), suggesting room for improvement under certain conditions.

**Table 4.** ANOVA results for thermal stability of PLA/surface-treated hemp fiber composites.

Source	Sum of squares	*df*	Mean square	*F*-value	*p*-value	
Model	7189.69	13	553.05	8.15	<0.0001	Significant
A-volume fraction	4313.51	1	4313.51	63.60	<0.0001	
B-print velocity	232.39	1	232.39	3.43	0.0806	
C-nozzle diameter	644.58	2	322.29	4.75	0.0220	
D-printing direction	400.76	2	200.38	2.95	0.0777	
AC	856.63	2	428.32	6.31	0.0084	
AD	352.54	2	176.27	2.60	0.1020	
BC	400.41	2	200.21	2.95	0.0779	
AÂ²	274.77	1	274.77	4.05	0.0593	
Residual	1220.89	18	67.83			
Lack of fit	1213.07	13	93.31	59.69	0.0001	Significant
Pure error	7.82	5	1.56			
Cor total	8410.57	31				
Std. Dev.	8.24		*R*²		0.8548	
Mean	317.83		Adjusted *R*²		0.7500	
C.V. %	2.59		Predicted *R*²		0.4778	
			Adeq precision		10.3136	

PLA: polylactic acid; ANOVA: analysis of variance.

The coded equation for predicting thermal degradation temperature is as follows:

(3) $$\begin{array}{rl} & \mathrm{Thermal}\;\mathrm{Degradation}\;\mathrm{Temperature}\;=\;+313.07\;-\;13.77\;A\;-\;3.99\;B\;-\;6.43\;C[1]\\ & \;\;\;\;\;\;\;\;\;\;\;\;\;\;\;\;\;\;\;\;\;\;\;\;\;\;\;\;\;\;\;\;\;\;\;\;\;\;\;\;\;\;\;\;\;\;\;\;\;\;\;\;\;\;\;\;\;\;\;\;\;\;\;\;\;\;\;\;\;\;\;\;\;\;+5.33\;C[2]\;+4.78\;D[1]-\;2.36\;D[2]-\;9.42\;\mathrm{AC}[1]\\ & \;\;\;\;\;\;\;\;\;\;\;\;\;\;\;\;\;\;\;\;\;\;\;\;\;\;\;\;\;\;\;\;\;\;\;\;\;\;\;\;\;\;\;\;\;\;\;\;\;\;\;\;\;\;\;\;\;\;\;\;\;\;\;\;\;\;\;\;\;\;\;\;\;\;\;\;+7.49\;\mathrm{AC}[2]\;+4.53\;\mathrm{AD}[1]\;-\;6.64\;\mathrm{AD}[2]\\ & \;\;\;\;\;\;\;\;\;\;\;\;\;\;\;\;\;\;\;\;\;\;\;\;\;\;\;\;\;\;\;\;\;\;\;\;\;\;\;\;\;\;\;\;\;\;\;\;\;\;\;\;\;\;\;\;\;\;\;\;\;\;\;\;\;\;\;\;\;\;\;\;\;\;\;\;-\;7.15\;\mathrm{BC}[1]+3.39\;\mathrm{BC}[2]+8.11\;{\tilde{A}}^{2} \end{array}$$

**Figure 6.**
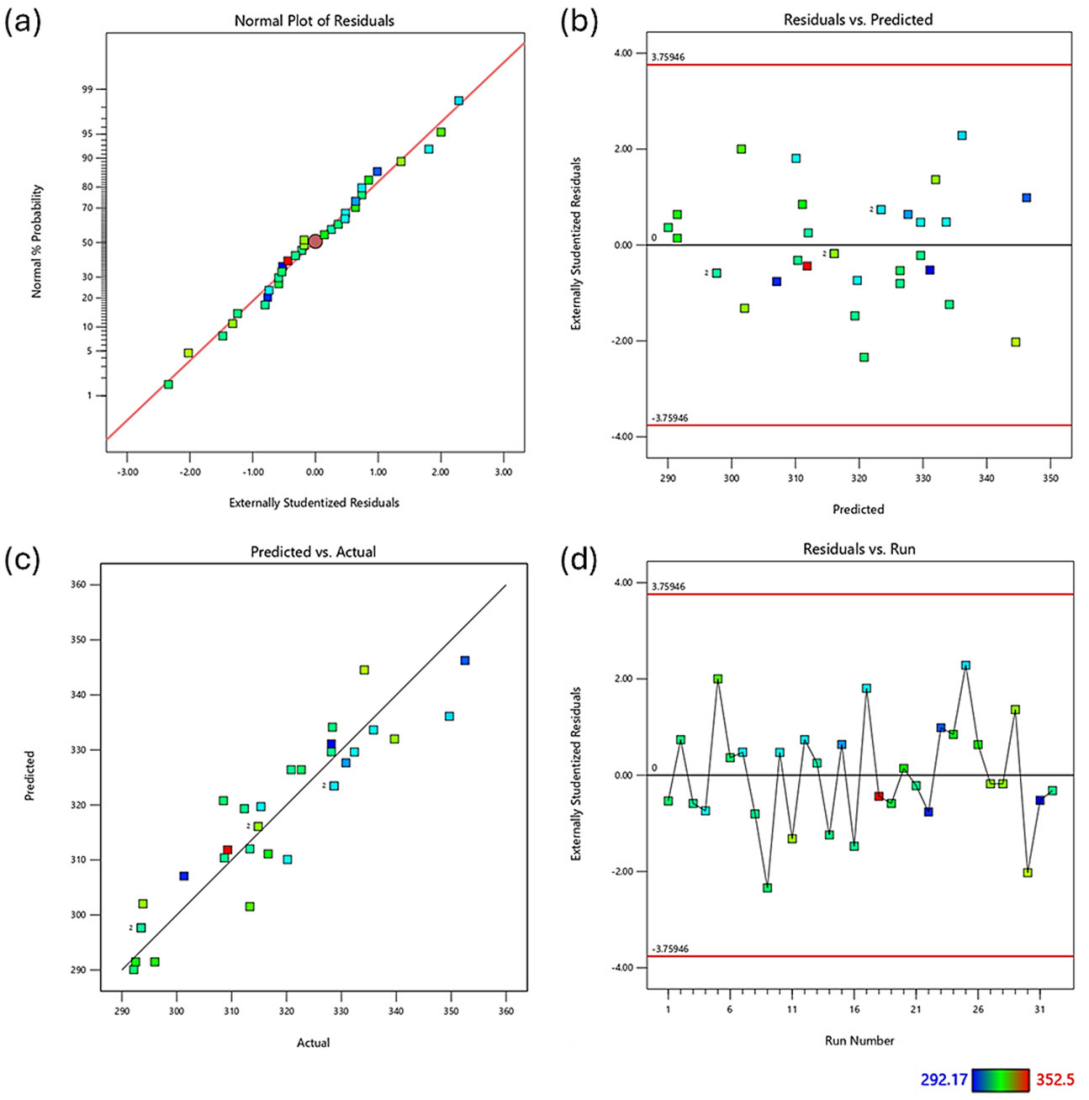
Diagnostic plots for regression analysis of thermal degradation temperature of polylactic acid (PLA)-HEMP composites. (a) Normal probability plot of residuals; (b) residuals vs. predicted values; (c) predicted vs. actual values; (d) residuals vs. run number. Color scale indicates thermal degradation temperatures ranging from 292.17 to 352.5 °C. These plots assess the model's adequacy in predicting the thermal stability of the composites for automotive applications.

This equation provides a quantitative framework for assessing the thermal degradation temperature based on the given levels of each factor, aiding in the optimization of composite materials for enhanced thermal stability in automotive applications. Diagnostic plots (Figure 6) provide insights into the model's performance. The normal probability plot of residuals (Figure 6(a)) shows general adherence to normality, with slight deviations at the extremes, indicating mostly normally distributed residuals. The residuals vs. predicted plot (Figure 6(b)) exhibits some patterning, suggesting potential areas for model improvement. The predicted vs. actual plot (Figure 6(c)) demonstrates a moderate correlation between predicted and measured values, reflecting the model's explanatory power while highlighting areas of uncertainty. Meanwhile, the residuals vs. run plot (Figure 6(d)) reveals some clustering, indicating possible time-related or run-order effects on the response.

**Figure 7.**
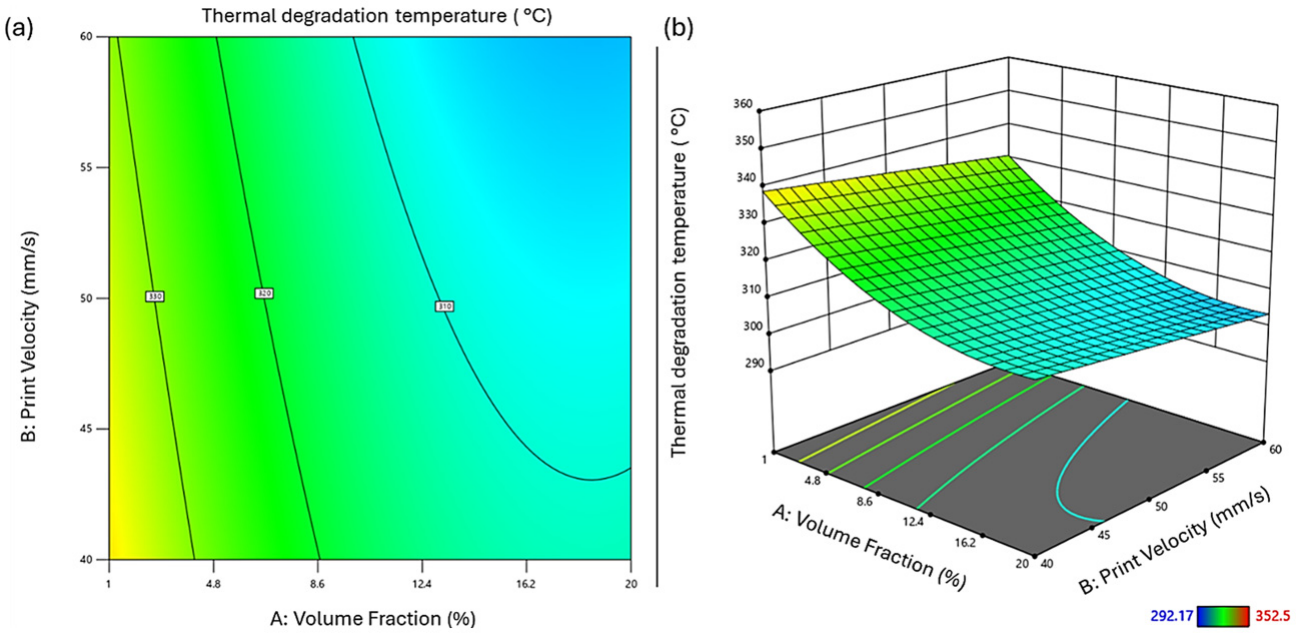
Influence of volume fraction and print velocity on the thermal degradation temperature of PLA/HEMP composites. (a) 2D contour plot and (b) 3D surface plot illustrating the relationship between volume fraction of HEMP fibers (A), print velocity (B), and thermal degradation temperature. The color gradient represents the thermal degradation temperature range from 292.17 °C (blue) to 352.5 °C (red). PLA: polylactic acid; 3D: three-dimensional; 2D: two-dimensional.

Analysis of individual factors and their interactions revealed that Vf (A), nozzle diameter (C), and their interaction (AC) are statistically significant (*p* < 0.05). The Vf demonstrated the most substantial impact on thermal degradation temperature, with an *F*-value of 63.60 (*p* < 0.0001). This finding underscores the critical role of fiber content in determining the thermal stability of PLA/HEMP composites. The 2D contour and 3D surface plots (Figure 7) illustrate the combined effects of Vf and print velocity on thermal degradation temperature. A pronounced decline in degradation temperature was observed with increasing fiber content, explained by the earlier thermal breakdown of hemicellulose and residual lignin in hemp. These components decompose below 300 °C and catalyze the degradation of PLA via char formation or volatile evolution. The trade-off between reinforcement and thermal resistance necessitates careful fiber loading design, especially for thermally demanding automotive environments.

The nozzle diameter and raster angle influenced thermal stability through their effects on flow and bonding. Smaller nozzles resulted in higher melt shear, improving polymer-fiber dispersion and potentially reducing voids, but may also promote fiber breakage and exposure, accelerating degradation. In contrast, larger nozzles provided more stable fiber encapsulation, reducing direct thermal exposure.

At a 45° raster angle, the thermal degradation temperature increased modestly due to enhanced heat distribution and minimized localized stress. The AC interaction term indicates that nozzle diameter modulates the effect of fiber volume, suggesting a synergistic influence on heat flow and material breakdown pathways.

Figure S3 presents the influence of Vf (A) and print speed (B) on the thermal degradation temperature of PLA reinforced with surface-treated hemp composites under different processing conditions.

Additionally, print velocity had a secondary but meaningful effect. Slower velocities allowed longer dwell times and better fusion, limiting oxygen ingress and thermal initiation sites. Rapid printing, however, trapped residual stresses and microvoids, acting as nucleation points for degradation. Overall, the model indicates that thermal stability is highest at low fiber contents, moderate velocities, and optimal nozzle/raster combinations. These findings enable strategic process adjustments to meet the specific thermal and mechanical demands of automotive components.

### Optimization, desirability, validation analysis

The desirability function analysis (DFA) was employed to optimize the mechanical and thermal properties of PLA/surface-treated hemp fiber composites intended for automotive applications. This method involves transforming individual responses into a scale-free desirability value ranging from 0 to 1, where 1 represents an entirely desirable outcome and 0 represents an entirely undesirable one. The overall desirability, *D*, is then calculated using the geometric mean of the individual desirability's, as shown below:

(4) $$D={\left[\overset{N}{\underset{\;j=1}{\prod }}\;di\right]}^{1/\sum ri}$$

(5) $$d_{i}(\overset{\wedge }{Y_{i}})=\left\{ \begin{array}{c} 0\\ {\left(\frac{{\overset{\wedge }{Y}}_{i}(x)-L_{i}}{T_{i}-L_{i}}\right)}^{s}\;if\;\;\overset{\wedge }{\underset{i}{Y}}\;(x)<L_{i},\;\\ 1 \end{array}\right.if\;\;L_{i}\leq \overset{\wedge }{\underset{i}{Y}}\;(x)\leq T_{i},\;if\;\;\overset{\wedge }{Y}\;(x)>T_{i}$$

where *N* is the number of responses, *d_j_* is the individual desirability for each response, and *r_i_* is the weight of *d_j_*. Different desirability functions are applied depending on whether a specific response is to be maximized, minimized, and targeted to a particular value.

**Figure 8.**
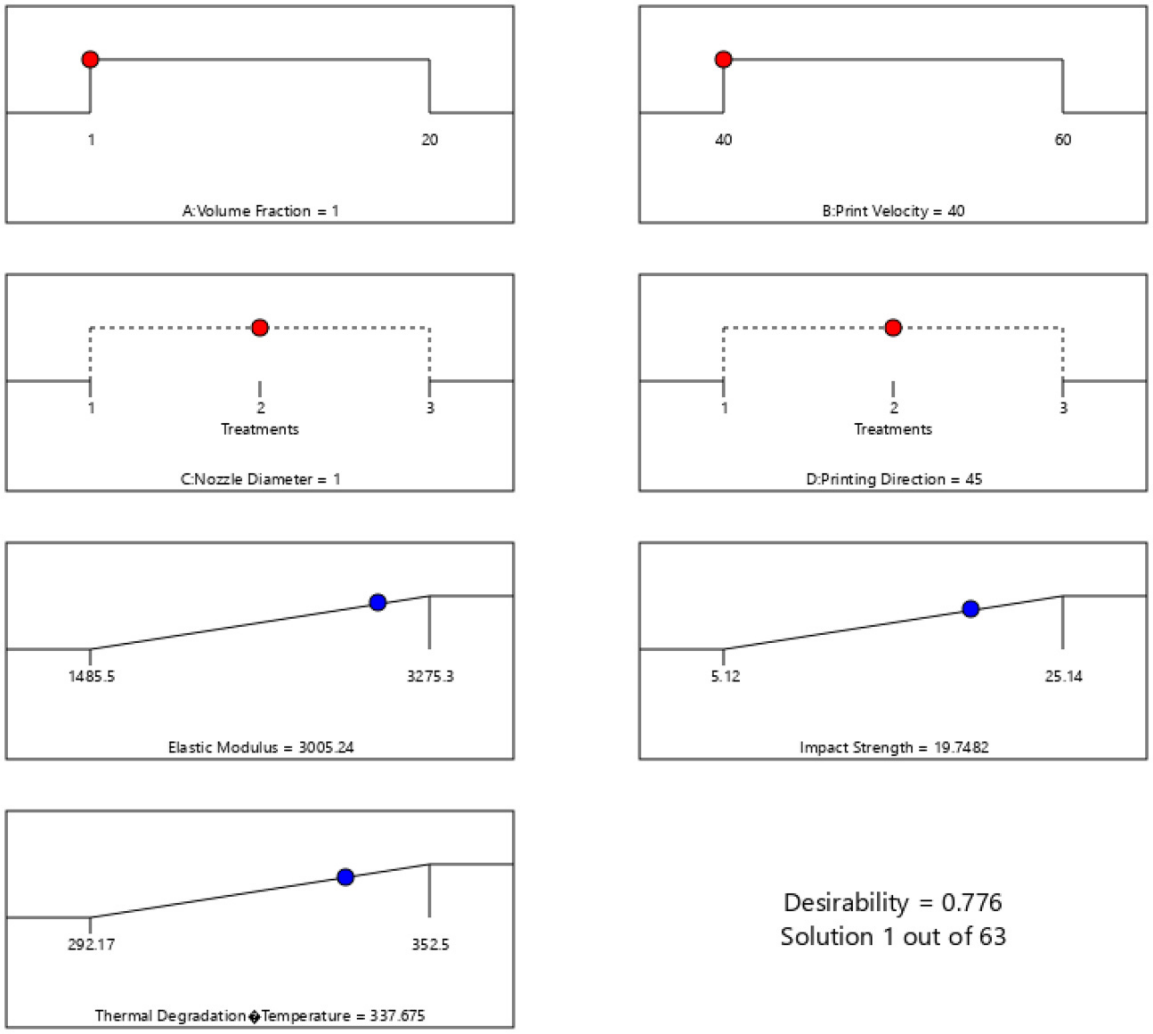
Ramp function graph showing the optimized response values based on the overall desirability of factor combination.

In this study, the responses were assigned equal weight and importance due to their critical role in the performance of the composite for automotive applications. The optimization process sought to maximize the elastic modulus, impact strength, and thermal degradation temperature. The optimal factor combination, achieving a desirability of 0.78, included a Vf of 1%, print velocity of 40 mm/s, nozzle diameter of 1 mm, and printing direction of 45° (Table S4). This combination led to the following optimized responses: elastic modulus of 3005.24 MPa, impact strength of 19.75 kJ/m^2^, and thermal degradation temperature of 337.68 °C (Figures 8 and S4).

**Figure 9.**
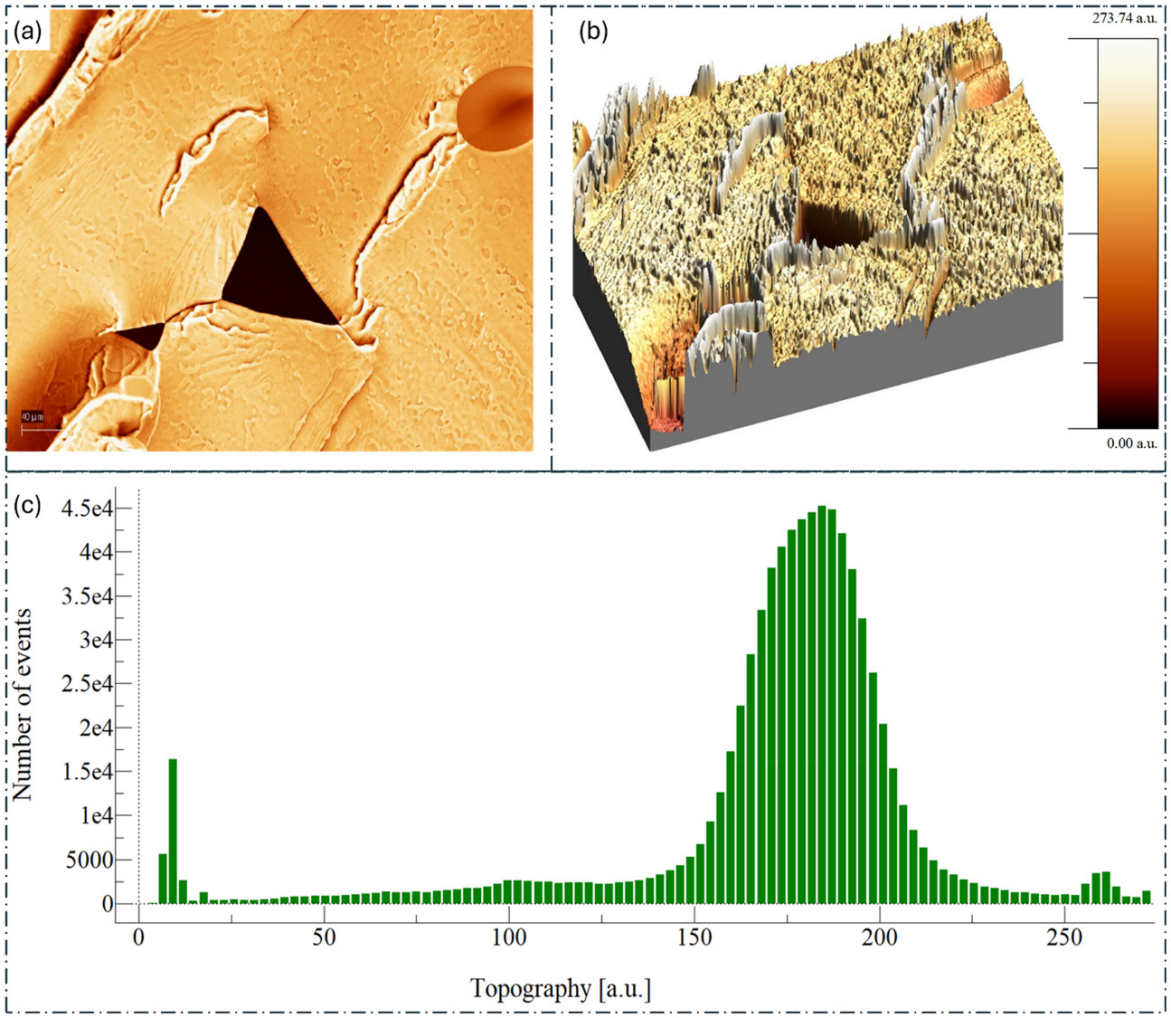
AFM surface topography and roughness analysis of PLA. (a) AFM micrograph showing the surface features of the PLA. (b) 3D surface topography visualization, illustrating the height variations and surface morphology of PLA. (c) Histogram depicting the distribution of topographical heights across the sample surface. PLA: polylactic acid; 3D: three-dimensional.

The contour and surface plots (Figure 9) further depict the effect of Vf and print velocity on the elastic modulus, demonstrating that higher Vfs and medium to high print velocities yield the highest elastic modulus values. To validate the model's predictive accuracy, confirmatory experiments were conducted across the design space, focusing, particularly on extreme conditions. Table 5 presents the outcomes of these confirmatory experiments results, validating the predictive model's performance for optimizing the thermal and mechanical properties of PLA reinforced with surface-treated hemp composite, focusing on automotive applications. The Observed response results in Table 5 reveal that the predicted mean and median values for the elastic modulus, impact strength, and thermal degradation temperature closely match the model's prediction in Table 6.

**Table 5.** Validation of the predictive model for optimized polylactic acid (PLA) reinforced with surface-treated HEMP composite.

	Factor 1	Factor 2	Factor 3	Factor 4	Response 1	Response 2	Response 3
	A: Volume fraction	B: Print velocity	C: Nozzle diameter	D: Printing direction	Elastic modulus	Impact strength	Thermal degradation temperature
Run	%	mm/s	mm	Â°	MPa	kJ/m^2^	°C
1	1	40	1	45	2882.50 (±149)	14.77 (±1.3)	334.17
2	1	40	1	45	2614.80 (±151)	16.23 (±1.6)	328.05
3	1	40	1	45	2333.90 (±182)	18.33 (±1.8)	335.02
4	1	40	1	45	2599.70 (±184)	17.31 (±0.8)	328.88
Mean					2607.25 (±224)	16.66 (±1.5)	331.53 (±3.6)

**Table 6.** Predicted and observed response values for the optimized PLA/surface-treated hemp fiber composite.

Solution 1 of 63 response	Predicted mean	Predicted median	Std Dev	*n*	SE Pred	95% PI low	Data mean	95% PI high
Elastic modulus (MPa)	3005.24	3005.24	188.197	3	203.187	2574.51	2607.73	3435.98
Impact strength (kJ/m^2^)	19.7482	19.7482	1.90607	3	2.09213	15.3131	16.66	24.1833
Thermal degradation temperature (°C)	337.675	337.675	8.23572	3	9.1027	318.551	331.585	356.799

PLA: polylactic acid; PI: prediction interval.

These results (Table 6) present the validation for key performance indicators: Elastic modulus, impact strength, and thermal degradation temperature. Statistical analyses include predicted means, standard deviation, sample size (*n*), standard error of prediction (SE Pred), and 95% prediction intervals (PIs).

Notably, the observed means fall within the 95% PIs, affirming the model's robustness in predicting the performance of the PLA/hemp fiber composite.

For the elastic modulus, the predicted mean is 3005.24 MPa, with a 95% PI ranging from 2574.51 to 3435.98 MPa. The observed data mean of 2607.25 MPa falls within this interval, indicating good model prediction. The impact strength shows a predicted mean of 19.7482 kJ/m^2^, with a 95% PI from 15.3131 to 24.1833 kJ/m^2^. The observed data mean of 16.66 MPa is also within this range, further validating the model's accuracy. For thermal degradation temperature, the predicted mean is 337.675 °C, with a 95% PI spanning from 318.551 to 356.799 °C. The observed data mean of 331.53 °C falls comfortably within this interval, supporting the model's predictive capability. These validation results demonstrate that the developed models adequately predict the effective elastic modulus, impact strength, and thermal degradation temperature of the optimized PLA reinforced with surface-treated HEMP composite. The observed means falling within the 95% PIs for all responses indicate the models’ reliability for predicting these key performance indicators in automotive applications.

In all, the confirmatory runs validate the adequacy of the models in predicting the elastic modulus, impact strength, and thermal degradation temperature of the optimized composite. This validation underscores the model's applicability in guiding the design and manufacturing process of PLA/surface-treated hemp fiber composites for automotive applications, ensuring that the desired mechanical and thermal properties are consistently achieved.

### Characterization of the optimized PLA/surface-treated hemp fiber composite

#### Morphological analysis of the optimized PLA/surface-treated hemp fiber composite

**Figure 10.**
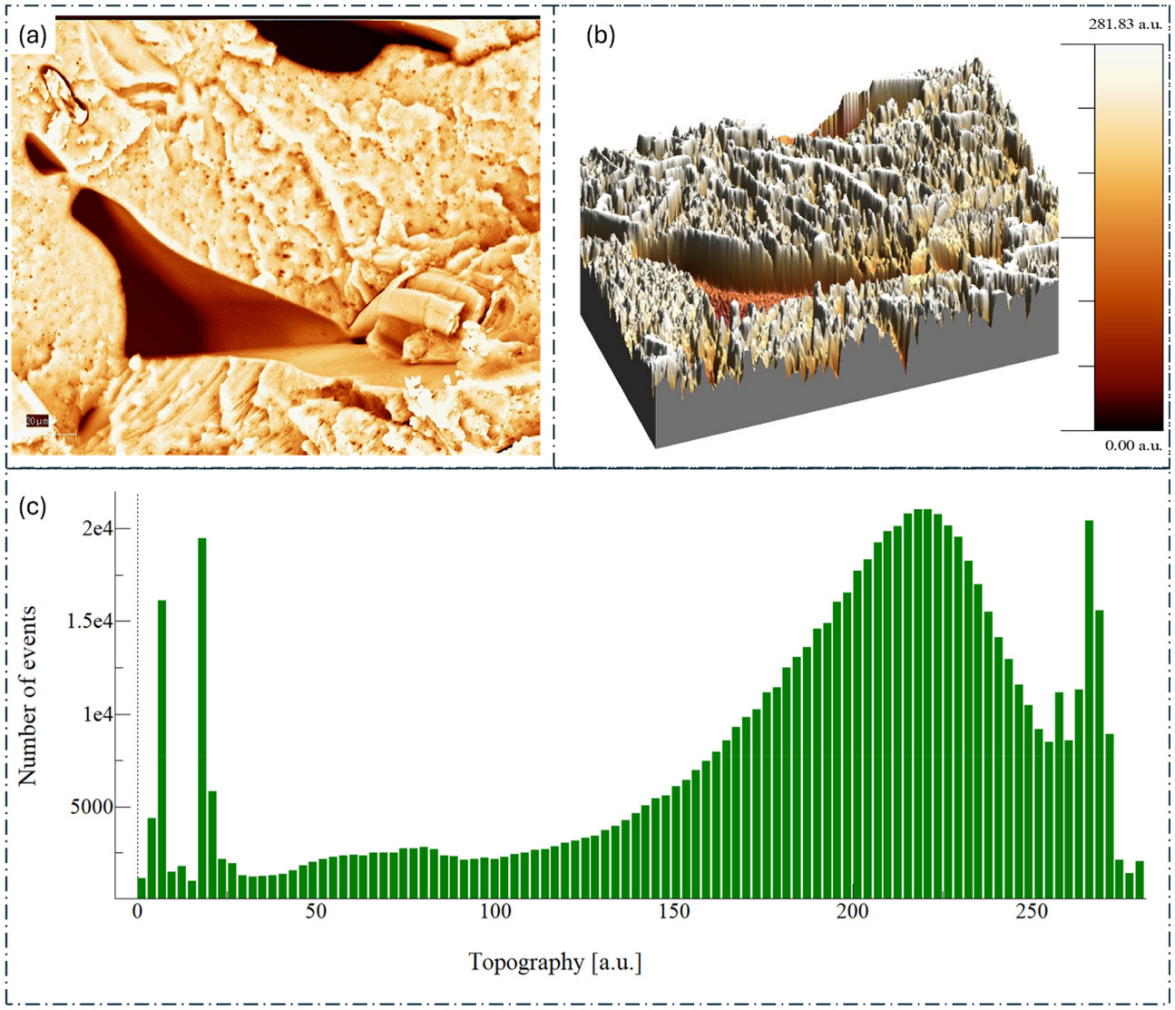
Surface topography and roughness analysis of PLA reinforced with surface-treated hemp. (a) 2D topographical map showing the surface features of the PLA/hemp composite. (b) 3D surface topography visualization, illustrating the height variations and surface morphology. (c) Histogram depicting the distribution of topographical heights across the sample surface. PLA: polylactic acid; 3D: three-dimensional; 2D: two-dimensional.

**Figure 11.**
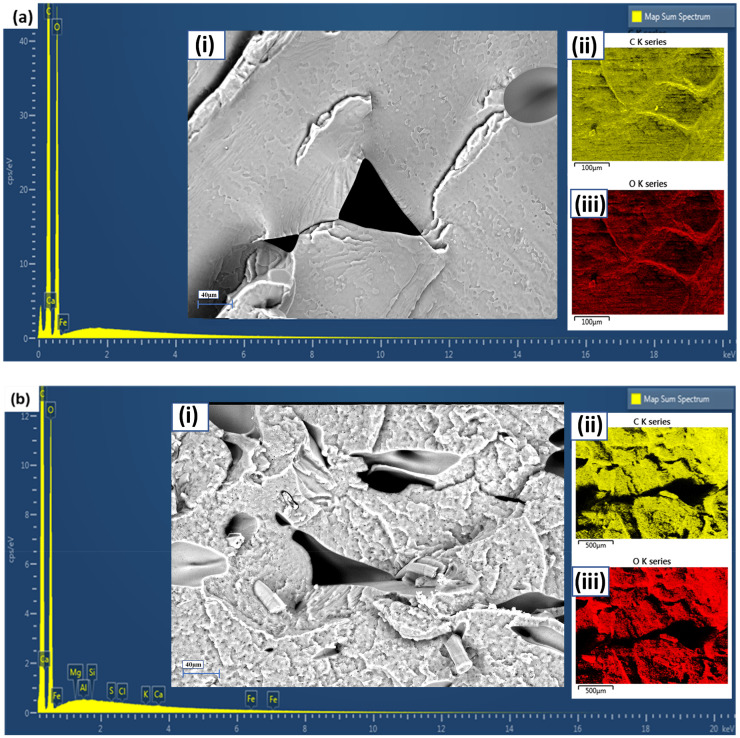
Elemental analysis and microstructure characterization of (a) PLA and (b) optimized surface-treated hemp fiber reinforced PLA composite. EDX spectrum of the composite, revealing the elemental composition. (i) SEM micrograph. (ii) C K-series map. (iii) O K-series map. Scale bars: 100 μm (i), 500 μm (ii, iii). PLA: polylactic acid; SEM: scanning electron microscopy; EDX: energy-dispersive X-ray analysis.

The morphological analysis of the optimized PLA/surface-treated hemp fiber composite compared to pure PLA reveals significant changes in surface characteristics and structure, as evidenced by the AFM and SEM with energy-dispersive X-ray analysis (EDX) results presented in Figures 9, 10, and 11. The AFM images provide valuable insights into the surface topography of both materials at the nanoscale. For pure PLA (Figure 8), the surface exhibits a relatively smooth topography with some visible features and irregularities. The RMS roughness of 44.41 nm indicates a moderately smooth surface, while the peak-to-peak height variation of 273.74 nm suggests the presence of notable surface features. The surface skewness of −1.93 points to an asymmetric height distribution skewed towards lower heights, and the surface kurtosis of 7.86 indicates a distribution with sharp peaks.

In contrast, the optimized PLA/hemp composite (Figure 10) displays a more textured and rougher surface compared to pure PLA. This is evident from the increased RMS roughness of 69.45 nm, signifying a substantial rise in surface roughness. The peak-to-peak height variation is slightly higher at 281.83 nm, further supporting the increased surface complexity. Interestingly, the surface skewness of −1.16, while still negative, is closer to zero, suggesting a more balanced height distribution. Additionally, the surface kurtosis decreases to 3.57, indicating a more normal distribution of heights across the surface.

These AFM results collectively suggest that the incorporation of surface-treated hemp fibers into the PLA matrix leads to a rougher, more textured surface. This increased roughness could potentially enhance mechanical interlocking and adhesion in subsequent processing applications, contributing to the improved mechanical properties observed in the optimized composite. Moving to the SEM and EDX analysis presented in Figure 11, we observe further distinctions between pure PLA and the optimized PLA/hemp composite. The SEM micrograph of pure PLA (Figure 11(a)) shows a relatively smooth and homogeneous surface with some visible defects and pores. The corresponding EDX spectrum indicates the presence of carbon (C) and oxygen (O), as expected for PLA, with elemental maps showing a uniform distribution of these elements across the surface.

In contrast, the SEM micrograph of the optimized PLA/hemp composite (Figure 11(b)) reveals a more complex surface morphology with visible fibers and a rougher texture. The EDX spectrum for the composite shows additional peaks of magnesium (Mg), potassium (K), aluminum (Al), iron (Fe), sulfur (S), silicon (Si), and chlorine (Cl), likely corresponding to elements present in the hemp fibers and introduced during the surface treatment process. The C and O elemental maps for the composite can be attributed to the organic nature of hemp, while the presence of Ca was also expected, as it is a major component in most plants. The introduction of traces of Mg, Al, Fe, Si, Cl, and S suggests that there were some soil impurities in our fibers. Jasti and Biswas^46^ also reported a similar elemental composition (C, O, Ca, Mg, Al, K, Si) when characterizing industrial hemp cannabis, attributing the additional peaks to impurities and trace elements from the soil. This SEM-EDX analysis demonstrates that the incorporation of surface-treated hemp fibers significantly alters both the surface morphology and elemental composition of the composite. The presence of fibers creates a more heterogeneous surface structure, which could contribute to improved adhesion through mechanical keying, thus enhancing the mechanical properties observed in the optimized composite.

Essentially, the morphological analysis reveals that the addition of surface-treated hemp fibers to PLA results in increased surface roughness and texture, more complex surface topography, heterogeneous elemental distribution, and visible fiber inclusions in the polymer matrix. These changes in surface characteristics and morphology likely contribute to the enhanced mechanical and thermal properties of the optimized PLA/hemp composite, as they can lead to improved fiber-matrix adhesion, better stress transfer, and potentially increased nucleation sites for crystallization. The rougher surface may also provide benefits in certain applications where increased surface area properties are desired, making this composite a promising material for automotive and other industrial applications requiring high-performance, eco-friendly materials.

#### Fourier transform infrared spectroscopy analysis of the optimized PLA/surface-treated hemp fiber composite

**Figure 12.**
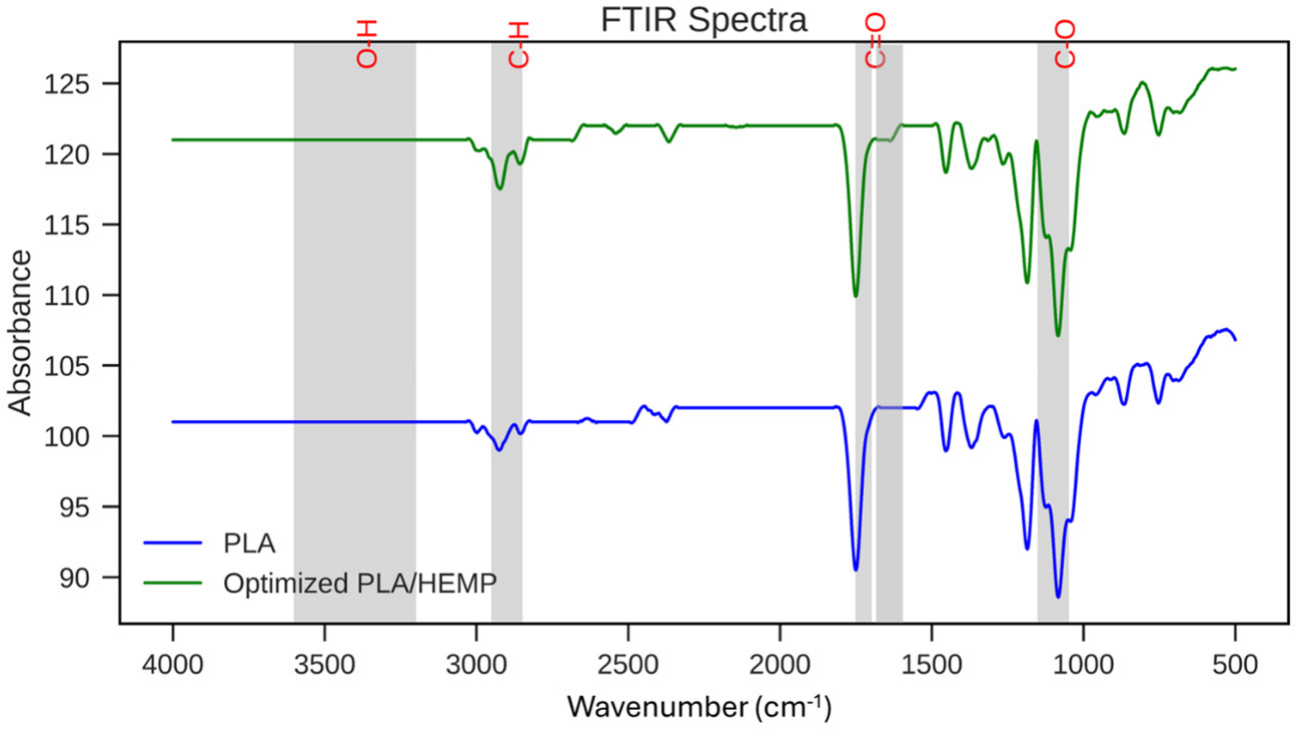
FTIR of the PLA and optimized PLA reinforced with surface-treated hemp composite. PLA: polylactic acid; FTIR: fourier transform infrared spectroscopy.

The fourier transform infrared spectroscopy (FTIR) spectra presented in Figure 12 provide valuable insights into the chemical structure and interactions of the optimized PLA/surface-treated hemp fiber composite when compared to pure PLA. This analysis helps elucidate the changes in functional groups and bonding that occur when surface-treated hemp fibers are incorporated into the PLA matrix. For pure PLA, the FTIR spectrum shows characteristic peaks that are typical of the polymer. A broad peak in the 3200–3600 cm^−1^ region indicates O-H stretching vibrations, likely corresponding to end groups in the PLA chains. Peaks in the 2800–3000 cm^−1^ range are attributed to C-H stretching of methyl and methylene groups in the PLA backbone. A strong peak around 1750 cm^−1^ is characteristic of the C=O stretching vibration of the ester groups in PLA. Multiple peaks in the 1000–1300 cm^−1^ region are associated with C-O stretching vibrations of the ester groups. This observation are similar to the finding that were reported by Cuiffo et al.^47^

When comparing the spectrum of the optimized PLA/HEMP composite to pure PLA, several notable differences and similarities can be observed. The O-H stretching region 3200–3600 cm^−1^ shows a broader and more intense peak for the composite. This increase in intensity and broadening can be attributed to the additional hydroxyl groups present in the cellulose and hemicellulose components of the hemp fibers. The C-H stretching region 2800–3000 cm^−1^ appears similar in both spectra, suggesting that the incorporation of hemp fibers does not significantly alter the aliphatic character of the material. The carbonyl C=O stretching peak around 1750 cm^−1^ remains prominent in the composite, indicating that the ester linkages of PLA are preserved. However, there may be slight shifts and changes in intensity due to potential interactions between the PLA matrix and the hemp fibers.

A new peak shoulder appears in the 1600–1680 cm^−1^ region for the composite, which is not present in pure PLA. This can be attributed to C=C stretching vibrations, likely from aromatic structures in lignin components of the hemp fibers. The C-O stretching region 1000–1300 cm^−1^ shows more complex and intense peaks in the composite, due to the overlapping C-O vibrations from both PLA and the cellulosic components of hemp fibers. The presence of these additional peaks and the changes in peak intensities in the composite spectrum provide evidence of successful incorporation of the hemp fibers into the PLA matrix. The broadening of the O-H peak suggests increased hydrogen bonding, which could contribute to improved interfacial adhesion between the fiber and matrix. This enhanced interaction is likely a result of the surface treatment applied to the hemp fibers, which can increase their compatibility with the PLA matrix. Similar characteristic peaks were reported by Mazzanti et al.^48^ and Ceylan et al.^49^ on the studies done on untreated and treated hemp reinforced composites.

The preservation of key PLA peaks in the composite spectrum indicates that the fundamental chemical structure of PLA is maintained. However, the subtle changes in peak shapes and intensities suggest that there are interactions between the PLA and hemp components, which could influence the material's properties. The appearance of new peaks associated with hemp fiber components confirms the presence of the fibers in the composite and provides information about their chemical nature post-treatment and processing.

Hence, this FTIR analysis demonstrates that the optimized PLA/surface-treated hemp fiber composite successfully combines the chemical characteristics of both PLA and hemp fibers. The spectral changes observed support the notion of improved interfacial interactions, which can contribute to the enhanced mechanical and thermal properties reported in other sections of the study. This analysis provides crucial molecular-level insights that complement the macroscopic property improvements observed in the optimized composite material, further validating the effectiveness of the optimization process in creating a high-performance, eco-friendly composite for automotive applications.

#### Mechanical properties of the optimized PLA/surface-treated hemp fiber composite

The mechanical properties of the optimized PLA/surface-treated hemp fiber composite found by confirmatory experiments in Table 6 were evaluated in comparison to pure PLA, focusing on elastic modulus and impact strength. Table 7 summarizes the key mechanical properties for both materials.

**Table 7.** Mechanical properties of pure polylactic acid (PLA) and optimized PLA/HEMP composite.

Material	Elastic modulus (MPa)	Impact strength (kJ/m^2^)	Thermal degradation temperature (°C)
Pure PLA	2353.27 (±109)	21.01 (±1.6)	334
Optimized PLA/HEMP composite	2607.25 (±224)	16.66 (±1.5)	331 (±3.6)

The optimized PLA/HEMP composite exhibited a significant enhancement in elastic modulus, with an increase of 10.79% when compared to pure PLA. This improvement can be attributed to several factors. Firstly, the incorporation of surface-treated hemp fibers provides additional stiffness to the PLA matrix, as hemp fibers typically possess a higher elastic modulus than PLA.^50^ Secondly, the sodium hydroxide and acetone treatment of hemp fibers likely improved fiber-matrix interfacial adhesion, allowing for more efficient stress transfer between the matrix and fibers.^21^ Additionally, the optimized printing direction (45°) may have aligned the fibers in a manner that maximizes their reinforcing effect. Similar improvement in elastic modulus were observed by Pokharel^51^ when characterizing PLA reinforced with hemp and flax fibers, respectively. The enhanced elastic modulus was attributed to improved adhesion occurring between the polymer and fibers at low loadings, restricting the chain movements and better stress transfer, Furthermore, as suggested by the differential scanning calorimetry (DSC) results, the presence of hemp fibers may have increased the overall crystallinity of the composite, which is often associated with increased stiffness in semi-crystalline polymers like PLA.^52^

Interestingly, the impact strength of the optimized PLA/HEMP composite decreased by 20.70% compared to pure PLA. This reduction can be explained by several factors. The inclusion of fibers, while enhancing stiffness, can create stress concentration points in the matrix, potentially leading to crack initiation under impact loads.^53^ Moreover, the presence of fibers may restrict the movement of polymer chains, reducing the overall ductility of the composite and its ability to absorb impact energy through plastic deformation. Although the surface treatment likely improved interfacial adhesion, it may not be optimal for transferring impact loads. Strong adhesion can sometimes lead to brittle failure modes, reducing impact resistance.^54^ Additionally, at the low fiber content of 1% Vf, the fibers may not provide sufficient toughening mechanisms (like fiber pull-out or crack bridging) to compensate for the reduced matrix ductility.

Compared to literature values, our optimized composite shows a 10.90% improvement in elastic modulus over a PLA/Hemp composite with 1% unmodified hemp reported by Coppola et al.,^55^ which had an elastic modulus of 2351 MPa. This enhancement is likely due to the surface treatment and optimized printing parameters employed in our study. The observed trade-off between increased stiffness and reduced impact resistance is a common phenomenon in composite materials.^54^ While the surface-treated hemp fibers significantly enhance the elastic modulus, they also introduce discontinuities in the PLA matrix that can act as stress concentrators, potentially reducing impact strength.

Therefore, the optimized PLA/surface-treated hemp fiber composite demonstrates a significant improvement in stiffness at the cost of impact resistance. This trade-off highlights the importance of carefully considering the desired property balance in the development of composites for specific automotive applications.

#### Thermal analysis of the optimized PLA/surface-treated hemp fiber composite

**Figure 13.**
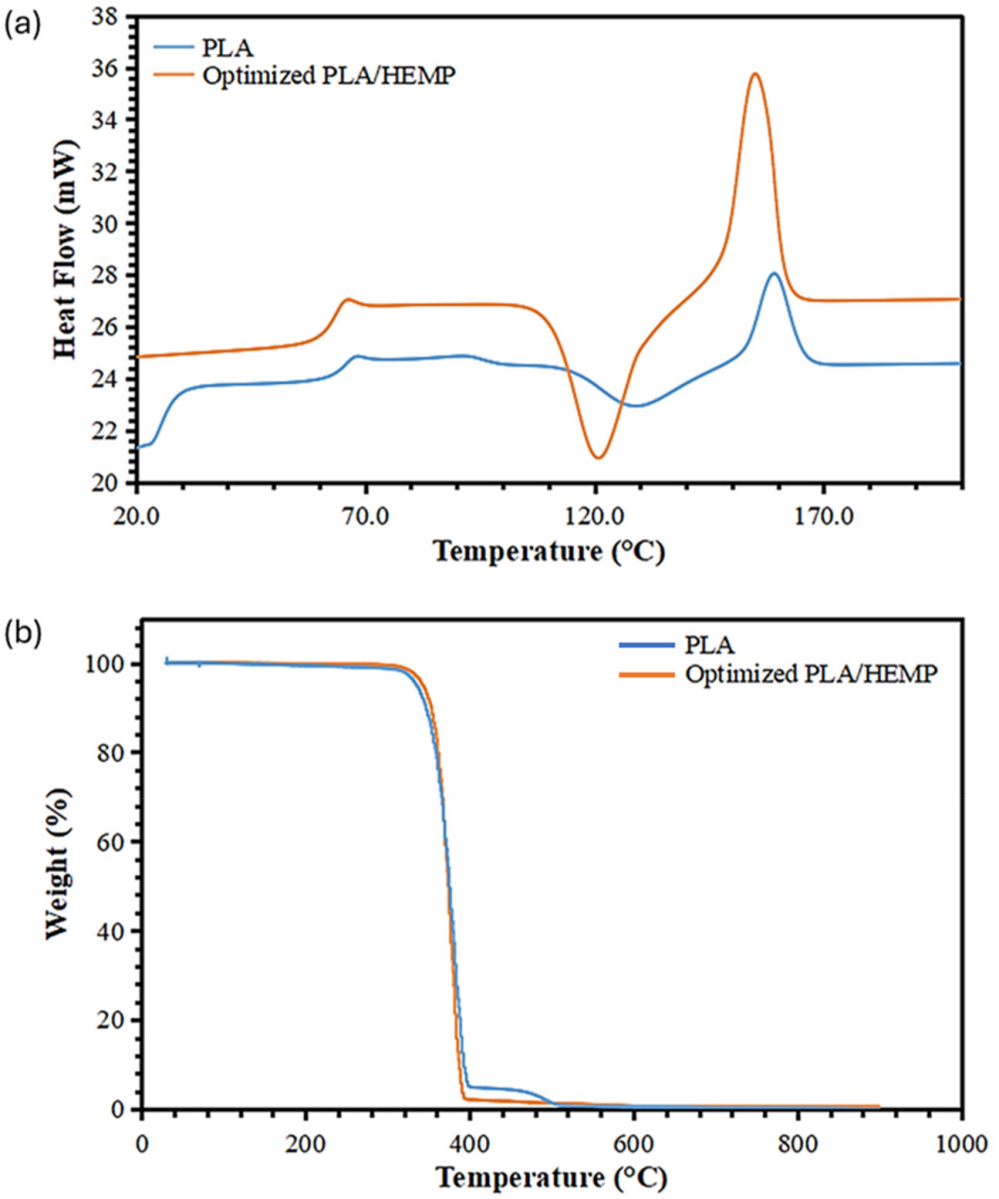
(a) DSC curve and (b) TGA depicting the thermal behavior of the PLA and optimized PLA reinforced with surface-treated hemp composite. PLA: polylactic acid; TGA: thermogravimetry analysis; DSC: differential scanning calorimetry.

The thermal analysis of the optimized PLA/surface-treated hemp fiber composite compared to pure PLA offers valuable insights into the thermal behavior and stability of these materials, as evidenced by the DSC and TGA results presented in Figure 13. Examining the DSC curves (Figure 13(a)), we observe significant differences in the thermal transitions and crystallization behavior between pure PLA and the optimized PLA/HEMP composite. For pure PLA, a glass transition is evident around 60 °C, indicated by a slight shift in the baseline. This is followed by a crystallization peak around 110–120 °C, and a subsequent melting peak at approximately 150–160 °C. These transitions are characteristic of semi-crystalline PLA and provide a baseline for comparison with the composite material.

In contrast, the optimized PLA/HEMP composite exhibits more pronounced and complex thermal behavior. The glass transition is less distinct, possibly due to the restricting effect of hemp fibers on polymer chain mobility. More notably, the crystallization peak is sharper and more intense, occurring at a slightly lower temperature, around 100–110 °C when compared to pure PLA. This suggests that the hemp fibers act as nucleation sites, promoting crystallization at lower temperatures and potentially increasing the overall crystallinity of the composite. Furthermore, the melting peak of the composite is significantly higher and sharper than that of pure PLA, indicating a higher degree of crystallinity and possibly more uniform crystal size distribution.

Transitioning to the TGA results (Figure 13(b)), the results gave insights into the thermal decomposition and stability of both materials, the results indicate that the materials are thermally stable upto temperatures above 150 °C. Pure PLA demonstrates a single-step degradation process, with the onset of significant weight loss occurring at 334 °C and completing by about 400 °C. This behavior is typical for PLA, which undergoes thermal decomposition through random chain scission. The optimized PLA/HEMP composite, however, exhibits a slightly different degradation profile. The onset of degradation appears to occur at a marginally lower temperature around 331 °C compared to pure PLA, which might be attributed to the earlier decomposition of some hemp fiber components and their interfacial regions with PLA.

Interestingly, the main degradation step of the composite seems to proceed at a slower rate, as evidenced by the less steep slope of the weight loss curve. This suggests that the incorporation of hemp fibers may provide some degree of thermal stabilization to the PLA matrix, possibly through char formation acting as a physical barrier to volatile decomposition products.

Another notable feature in the TGA curve of the composite is the presence of a small residual mass at high temperatures (>500 °C) that is not observed in pure PLA. This residue likely corresponds to the slow degradation of lignin and inorganic components and carbonaceous residues from the hemp fibers, indicating potential improvements in fire resistance properties.

These thermal analysis results have significant implications for the potential applications of the optimized PLA/HEMP composite in automotive and other industries. The enhanced crystallization behavior could lead to improved mechanical properties, particularly in terms of stiffness and heat resistance. The higher melting temperature of the composite may extend its usable temperature range in automotive applications, while the altered thermal degradation profile, despite showing earlier onset, demonstrates a more gradual decomposition process. This could potentially provide a wider processing window and better performance in high-temperature automotive environments.

## Conclusion

This research successfully used the combination of additive manufacturing and RSM to prepare the optimized PLA reinforced with surface-treated hemp composites for automotive applications with improved properties when compared to pure PLA. Significant improvement in mechanical and thermal properties was observed with the combination of 1% Vf, 40 mm/s print velocity, 1 mm nozzle diameter, and 45° printing direction. This combination yielded an elastic modulus of 2607.27 MPa, impact strength of 16.66 kJ/m^2^, and thermal degradation temperature of 331.53 °C. AFM and SEM images revealed an increased surface roughness and complexity, enhancing fiber-matrix adhesion. DSC and TGA analyses showed improved crystallization behavior with a lower onset temperature and higher melting point, indicating better thermal stability. This further affirms that these materials may perform well in interior automotive components that do not require high temperatures. The optimized PLA/hemp composite, with its enhanced mechanical properties, improved thermal stability resulting in gradual decomposition, and renewable resource base, aligns with the automotive industry's sustainability goals. This work provides a robust framework for developing high-performance, eco-friendly materials, utilizing a DFA with a score of 0.782. Future research should focus on scaling production, long-term performance testing under automotive conditions, and exploring additional surface treatments to further enhance the composite's properties for industrial applications.

### Limitations and recommendations

Despite the valuable insight, the study is limited to four variables. The significant lack of fit suggests that some variability in the data was not adequately captured by the model. This indicates the possible influence of additional factors or interactions affecting the mechanical properties that were not included in the current model, warranting further investigation in future studies. Moreover, future research could focus on examining the thermal behavior of NFRPCs to evaluate their performance under conditions exceeding their glass transition temperatures, as well as the effect of fiber aspect ratio. Additionally, the improvement in adhesion and reduction of micro-voids using optimized coupling agents should be investigated.

## Supplemental Material

sj-docx-1-sci-10.1177_00368504251352072 - Supplemental material for Thermal and mechanical properties of polylactic acid reinforced with surface-treated hemp composites for automotive applications Supplemental material, sj-docx-1-sci-10.1177_00368504251352072 for Thermal and mechanical properties of polylactic acid reinforced with surface-treated hemp composites for automotive applications by Sifiso J Skosana, Moshibudi C Khoathane, Thomas Malwela and Wilson Webo in Science Progress
