# Supplementary material for: Thermal and mechanical properties of polylactic acid reinforced with surface-treated hemp composites for automotive applications
Source: Sci Prog. 2025 Oct 16;108(4):00368504251352072. doi: 10.1177/00368504251352072 (PMC12536183; doi:10.1177/00368504251352072)
Supplement: sj-docx-1-sci-10.1177_00368504251352072 - Supplemental material for Thermal and mechanical properties of polylactic acid reinforced with surface-treated hemp composites for automotive applications [file sj-docx-1-sci-10.1177_00368504251352072.docx]

**SUPPLEMENTARY**

**Optimizing the Thermal and Mechanical Performance of** **PLA Reinforced with Surface-Treated HEMP Biocomposite for Automotive Applications**

Sifiso John Skosana^1^. Caroline Khoathane^1^. Thomas Malwela^2^. Wilson Webo^1^

^1^Department of Chemical, Metallurgical and Materials Engineering, Faculty of Engineering and the Built Environment, Tshwane University of Technology, Pretoria 0001, South Africa

­^2^Departmet of Physics, University of Limpopo, Private Bag X1106, Sovenga 0727, South Africa

Corresponding Author. Emails: thomas.malwela@ul.ac.za

**Table S1**: Parameters and characteristics of the Factors for the printing process

| **Factor** | **Name** | **Units** | **Type** | **SubType** | **Minimum** | **Maximum** | **Coded Low** | **Coded High** | **Mean** | **Std. Dev.** |
| --- | --- | --- | --- | --- | --- | --- | --- | --- | --- | --- |
| A | Volume Fraction | % | Numeric | Continuous | 1.00 | 20.00 | -1 ↔ 1.00 | +1 ↔ 20.00 | 11.01 | 7.47 |
| B | Print Velocity | mm/s | Numeric | Continuous | 40.00 | 60.00 | -1 ↔ 40.00 | +1 ↔ 60.00 | 49.55 | 7.55 |
| C | Nozzle Diameter | mm | Categoric | Nominal | 0.4 | 2 |  |  | **Levels:** | 3.00 |
| D | Printing Direction | ° | Categoric | Nominal | 0 | 90 |  |  | **Levels:** | 3.00 |

**Table** S2: Parameters and characteristics of the Reponses for the printing process

| **Response** | **Name** | **Units** | **Observations** | **Minimum** | **Maximum** | **Mean** | **Std. Dev.** | **Ratio** |
| --- | --- | --- | --- | --- | --- | --- | --- | --- |
| R1 | Elastic Modulus | MPa | 32.00 | 1485.5 | 3275.3 | 2192.85 | 371.54 | 2.20 |
| R2 | Impact Strength | MPa | 32.00 | 5.12 | 25.14 | 11.95 | 5.57 | 4.91 |
| R3 | Thermal Degradation  Temperature | ° C | 32.00 | 292.17 | 352.5 | 317.83 | 16.47 | 1.21 |

| **Table** S3: Constraints characteristics of the Factors and Responses for the printing process | | | | | | |
| --- | --- | --- | --- | --- | --- | --- |
| **Name** | **Goal** | **Lower Limit** | **Upper Limit** | **Lower Weight** | **Upper Weight** | **Importance** |
| A:Volume Fraction | is in range | 1 | 20 | 1 | 1 | 3 |
| B:Print Velocity | is in range | 40 | 60 | 1 | 1 | 3 |
| C:Nozzle Diameter | is in range | 0.4 | 2 | 1 | 1 | 3 |
| D:Printing Direction | is in range | 0 | 90 | 1 | 1 | 3 |
| Elastic Modulus | maximize | 1485.5 | 3275.3 | 1 | 1 | 3 |
| Impact Strength | maximize | 5.12 | 25.14 | 1 | 1 | 3 |
| Thermal Degradation Temperature | maximize | 292.17 | 352.5 | 1 | 1 | 3 |

**Table** S4: 5 of the 63 Solutions found for the desirability analysis.

| **Table S4: 5 of the 63 Solutions found for the desirability analysis.** | | | | | | | | | |
| --- | --- | --- | --- | --- | --- | --- | --- | --- | --- |
| **Number** | **Volume Fraction** | **Print Velocity** | **Nozzle Diameter** | **Printing Direction** | **Elastic Modulus** | **Impact Strength** | **Thermal Degradation**  **Temperature** | **Desirability** |  |
| **1** | **1.000** | **40.000** | **1** | **45** | **3005.242** | **19.748** | **337.675** | **0.776** | **Selected** |
| 2 | 1.289 | 40.000 | 1 | 45 | 3008.195 | 19.749 | 336.797 | 0.772 |  |
| 3 | 1.000 | 40.000 | 2 | 45 | 2760.173 | 21.582 | 338.644 | 0.767 |  |
| 4 | 1.000 | 40.081 | 2 | 45 | 2755.637 | 21.565 | 338.642 | 0.766 |  |
| 5 | 1.143 | 40.000 | 2 | 45 | 2761.642 | 21.583 | 338.122 | 0.764 |  |


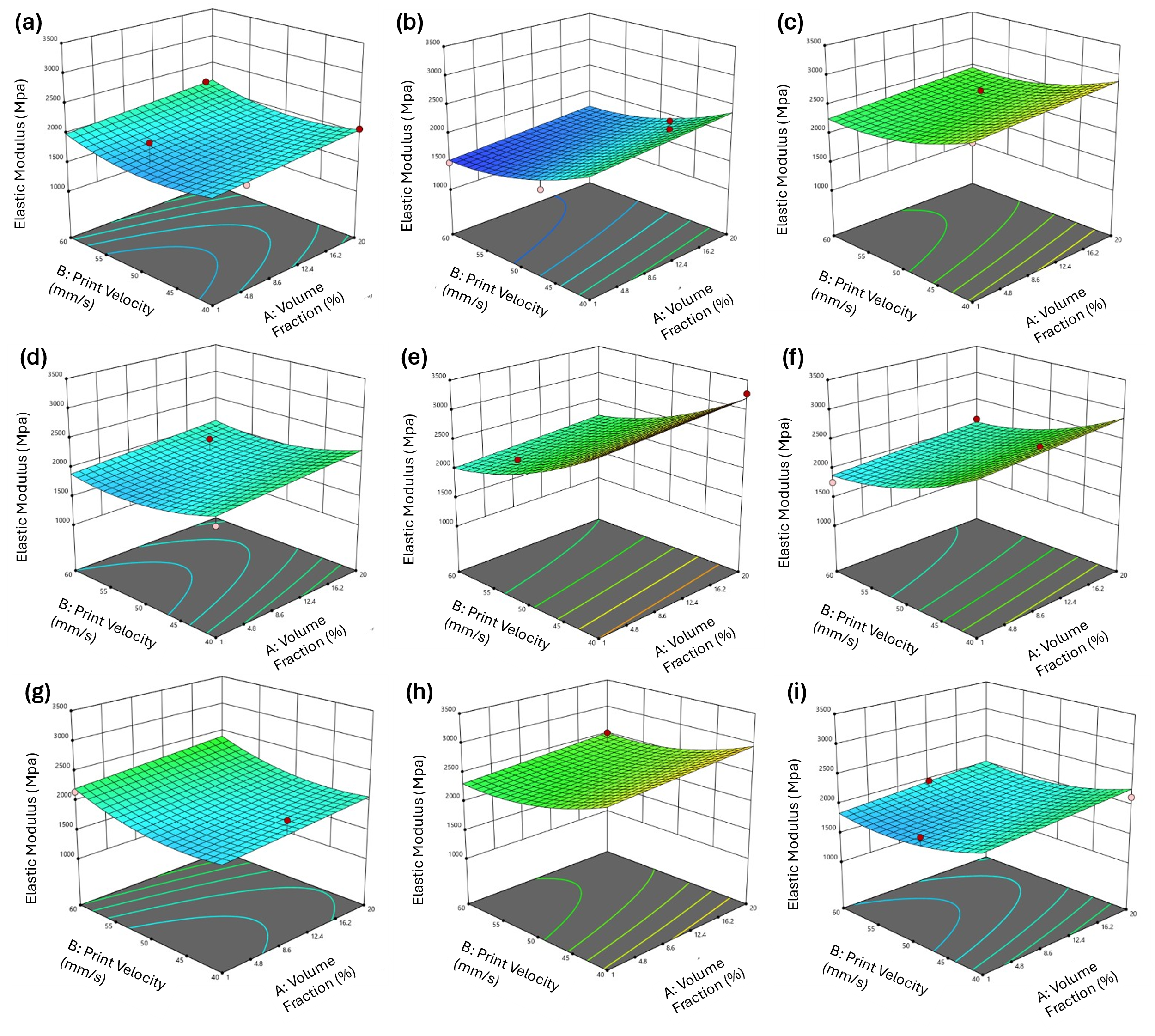


**Figure S1**: Response surface and contour plots illustrating the effects of volume fraction (A) and print velocity (B) on the elastic modulus of PLA reinforced with surface-treated hemp biocomposites under various processing conditions. Subplots (a-i) represent different combinations of nozzle diameter and printing direction: (a,d,g) 0.4 mm nozzle; (b,e,h) 1.0 mm nozzle; (c,f,i) 2.0 mm nozzle; (a,b,c) 0° printing direction; (d,e,f) 45° printing direction; (g,h,i) 90° printing direction. The color gradients and contour lines indicate the magnitude of the elastic modulus, with warmer colors representing higher values.


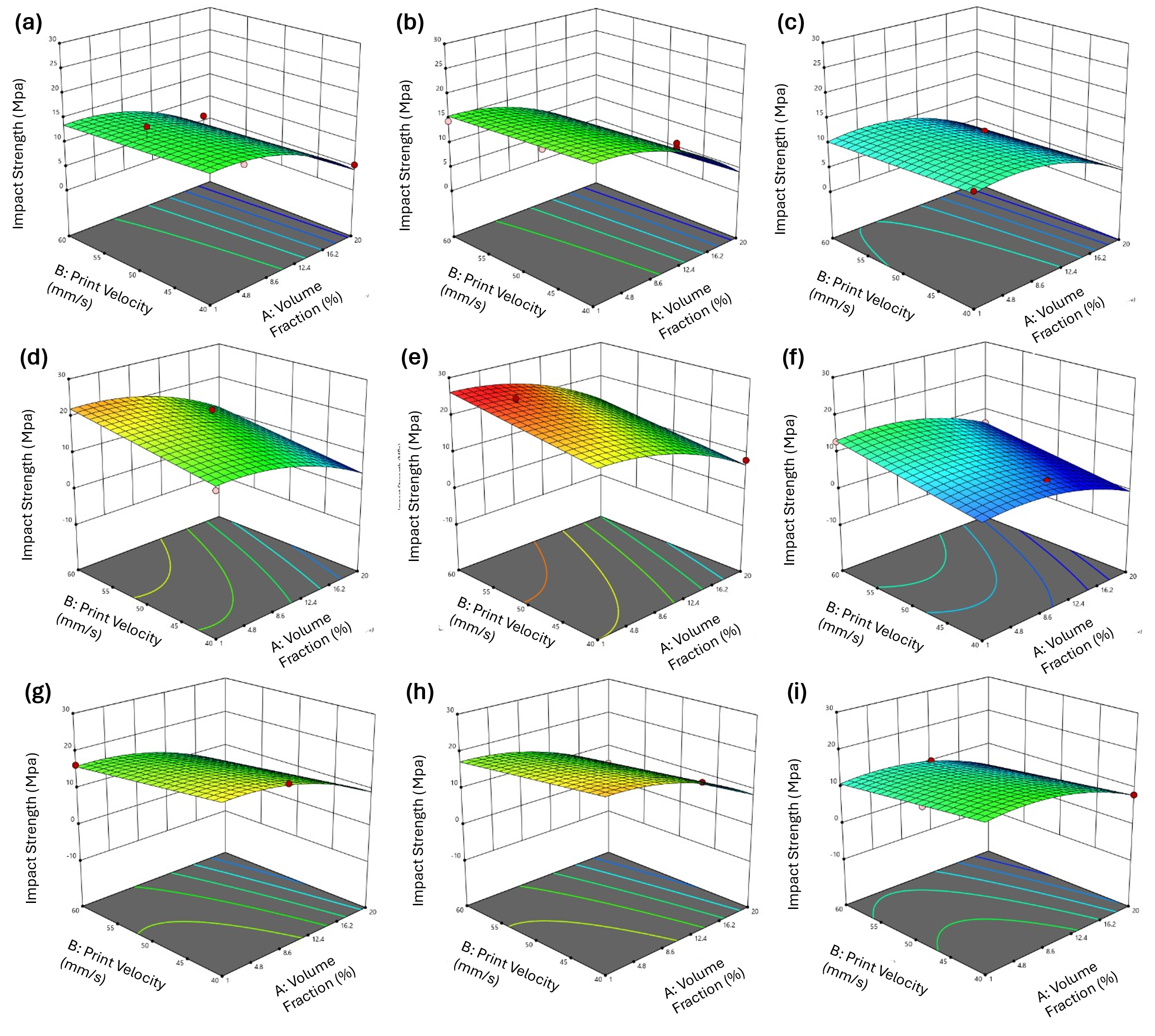


**Figure S2**: 3D contour plots illustrating the effects of volume fraction (A) and print velocity (B) on the impact strength of PLA reinforced with surface-treated hemp biocomposites under various processing conditions. Subplots (a-i) represent different combinations of nozzle diameter and printing direction: (a,d,g) 0.4 mm nozzle; (b,e,h) 1.0 mm nozzle; (c,f,i) 2.0 mm nozzle; (a,b,c) 0° printing direction; (d,e,f) 45° printing direction; (g,h,i) 90° printing direction. The color gradients and contour lines indicate the magnitude of the elastic modulus, with warmer colors representing higher values.


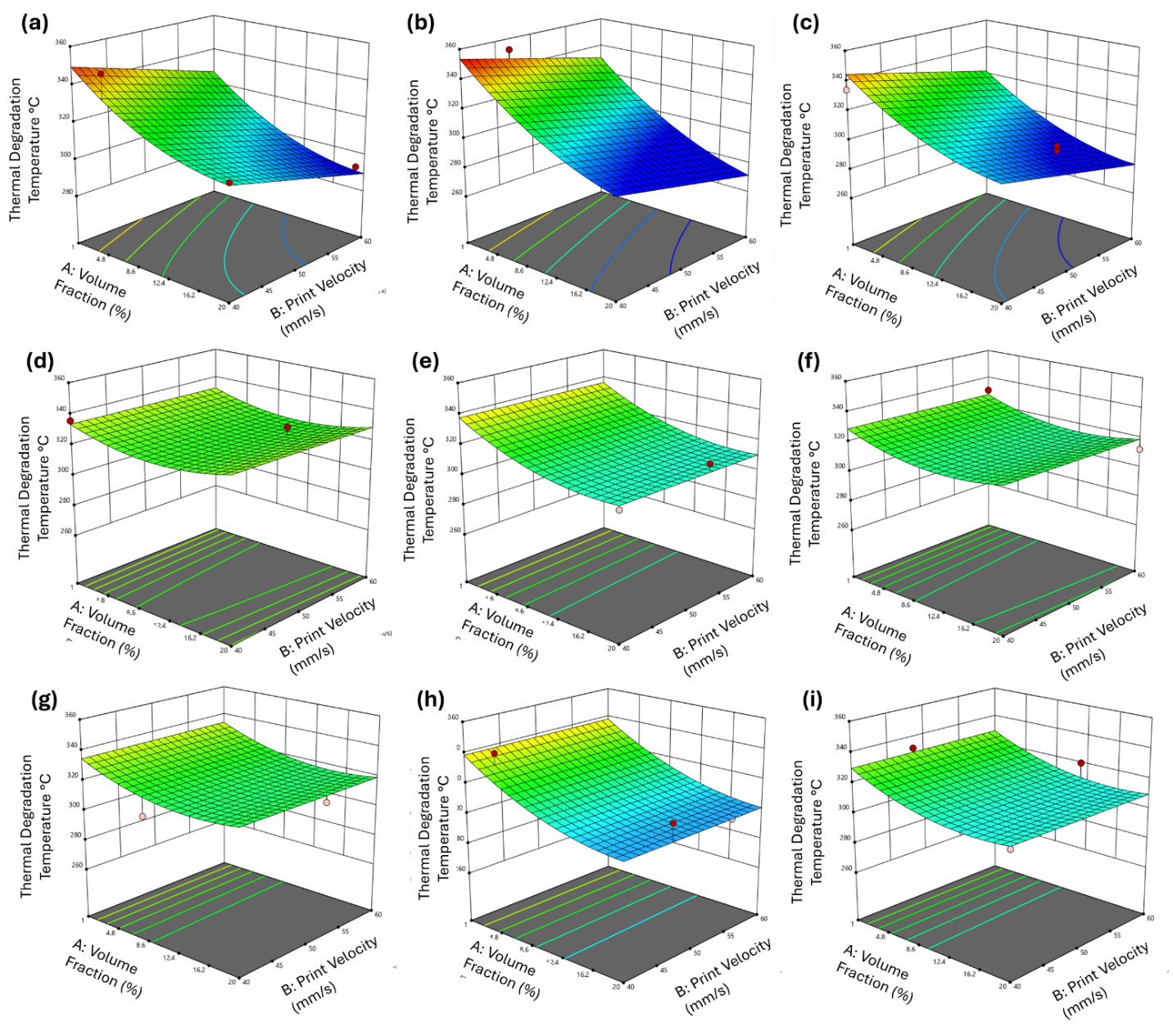


**Figure S3**: 3D contour plots illustrating the effects of volume fraction (A) and print velocity (B) on the thermal stability of PLA reinforced with surface-treated hemp biocomposites under various processing conditions. Subplots (a-i) represent different combinations of nozzle diameter and printing direction: (a,d,g) 0.4 mm nozzle; (b,e,h) 1.0 mm nozzle; (c,f,i) 2.0 mm nozzle; (a,b,c) 0° printing direction; (d,e,f) 45° printing direction; (g,h,i) 90° printing direction. The color gradients and contour lines indicate the magnitude of the elastic modulus, with warmer colors representing higher values.


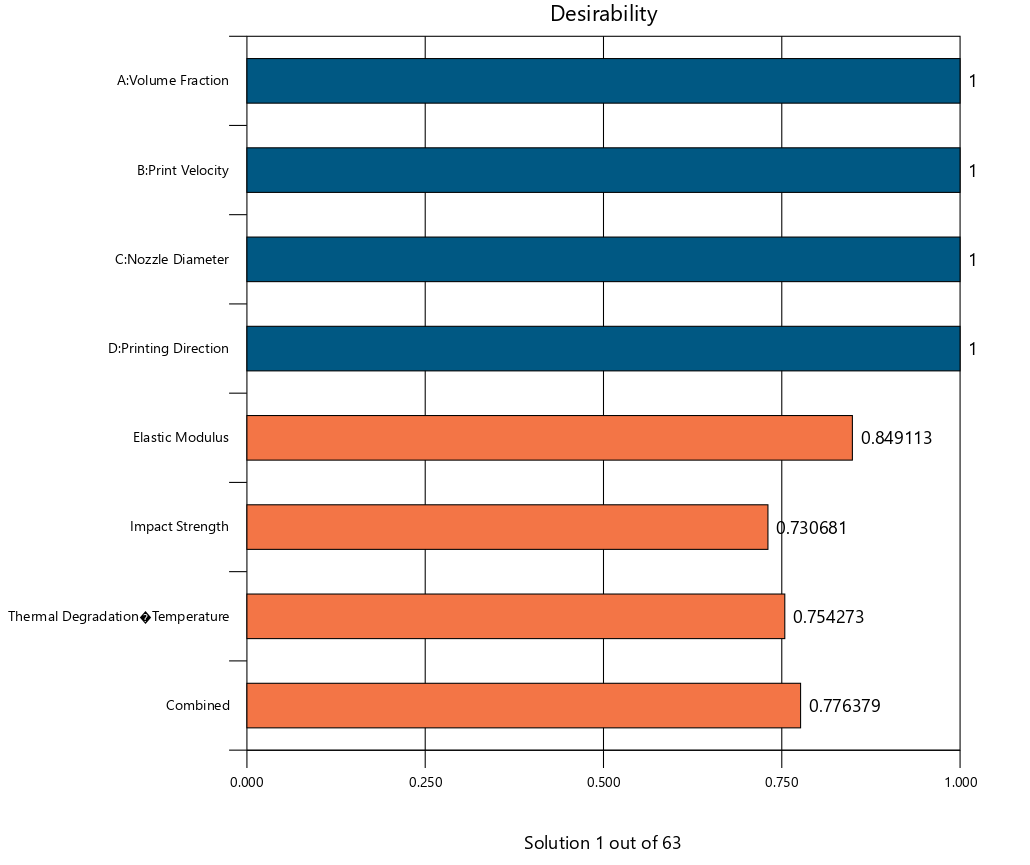


**Figure S4**: Desirability analysis for optimizing PLA/surface-treated hemp fiber composites. The graph shows the desirability scores for input factors (volume fraction, print velocity, nozzle diameter, and printing direction) and output responses (elastic modulus, impact strength, and thermal degradation temperature). The combined desirability score represents the overall optimization outcome for the composite material intended for automotive applications. Input factors show maximum desirability (1.0), while output responses and the combined score indicate the degree of optimization achieved for each property.
